# Supplementary material for: Vessels encapsulating tumor clusters predict better outcomes in advanced hepatocellular carcinoma treated with atezolizumab–bevacizumab
Source: JHEP Rep. 2026 Apr 29;8(7):101874. doi: 10.1016/j.jhepr.2026.101874 (PMC13276544; doi:10.1016/j.jhepr.2026.101874)

Vessels encapsulating tumor clusters predict better outcomes in advanced hepatocellular carcinoma treated with atezolizumab–bevacizumab

Alexandre Sayadi, Claudia Campani, Astrid Laurent-Bellue, Marianne Ziol, Luca Di Tommaso, Etienne Becht, Benoit Terris, Julien Calderaro, Luca Messerini, Miguel Albuquerque, Federico Lipsich, Carolina Gutierrez, Marion Dhooge, Lisa Lellouche, Giuliana Amaddeo, Alina Pascale, Olivier Rosmorduc, Fabio Marra, Lorenza Rimassa, Marco Dioguardi Burgio, Jean-Charles Nault, Mohamed Bouattour, Valérie Paradis, Aurélie Beaufrère

Table of contents

Supplementary methods 3
Supplementary references 4
Table S1 5
Table S2 6
Table S3 7
Table S4 7
Table S5 8
Table S6 10
Table S7 11
Table S8 13
Table S9 15
Table S10 17

Table S11 19

Fig. S1. 22

Fig. S2 23

Fig. S3 24

Fig. S4. 25

Fig. S5. 26

Fig. S6 27

Fig. S7 28

Fig. S8. 29

Fig. S9 30

Supplementary methods

Clinical data collection

The following baseline characteristics were collected: age, sex, height, weight, underlying liver disease etiology (HCV and/or HBV infection, Metabolic-dysfunction Associated Steatotic Liver Disease [MASLD], Alcohol-associated Liver Disease [ALD], Metabolic and Alcohol-associated Liver Disease [MetALD]), Eastern Cooperative Oncology Group (ECOG) Performance Status, presence of underlying cirrhosis and Child-Pugh class[1]. Tumor characteristics included macrovascular invasion, extrahepatic spread and BCLC stage. Prior locoregional or systemic treatment were also recorded. Baseline blood tests included bilirubin, international normalized ratio (INR), C-reactive protein (CRP), and alpha-fetoprotein (AFP) levels.

Immunohistochemistry

For Beaujon Hospital's cases in the discovery cohort, supplementary FFPE tissue sections were stained with the following antibodies, depending on remaining material: CD3 (DAKO, A0452, 1:100), CD8 (DAKO, C8/144B, M7103, 1:50), PD-L1 (QUARTETT, QR1, 1-PR292-02, 1:100), glutamine synthetase (GS, Chemicon, GS-6, MAB302, 1:200), β-catenin (BD Biosciences, 14/Beta-Catenin, 610153, 1:200) and p53 (DAKO, DO7, M700101, 1:100). Intratumoral CD8+ and CD3+ T cell density was determined as the density per mm² of CD8-stained cells occupying the tumor, using positive cell detection in QuPath software (v.0.5.0)[2].

Tumor proportion score (TPS) was defined as the number of PD-L1+ tumor cells divided by all tumor cells; whereas Combined Positive Score (CPS) was defined as the

number of PD-L1+ tumor cells and inflammatory cells (i.e., lymphocytes and macrophages) divided by all tumor cells. *CTNNB1* mutational status was inferred from diffuse and intense GS staining and/or nuclear β-catenin localization. *TP53* mutational status was inferred from strong, diffuse nuclear p53 staining or complete absence of staining.

Supplementary references

[1] Rinella ME, Lazarus JV, Ratziu V, et al. A multisociety Delphi consensus statement on new fatty liver disease nomenclature. Journal of Hepatology 79:1542-56.

[2] Bankhead P, Loughrey MB, Fernández JA, et al. QuPath: Open source software for digital pathology image analysis. Sci Rep 7. Available on: https://www.nature.com/articles/s41598-017-17204-5.

Table S1. Criteria for histopathological assessment

Criteria	Assessment
Histopathologic subtype (according to WHO)	**Steatohepatitic (SH):** Among the 5 following criteria (ballooning, Mallory's body, inflammation, fibrosis and steatosis); presence of at least 3 criteria on more than 50% of the tumor surface area
	**Macrotrabecular massive (MTM):** Macrotrabecular architecture (hepatic cord thickness >6 cells) over more than 50% of tumor surface area
	**Scirrhous (SQ):** Abundant fibrosis on more than 50% of the tumor surface, without criteria for SH-HCC
	**Clear cell (CC):** Tumor cells clarified on more than 80% of tumor surface area
	**Neutrophil-rich (NR):** Neutrophil-rich infiltrate covering more than 50% of the tumor surface, with no other predominant subtype
	**Lymphocyte-rich (LR):** Lymphocyte-rich infiltrate over 50% of tumor surface, with no other predominant subtype
	**Not Other Specified (NOS):** Exclusion diagnosis after elimination of subtypes listed above
VETC phenotype	- Non-VETC-HCC: No VETC images<br>- Incomplete VETC phenotype: Focal VETC images (1 - 54% of tumor surface)<br>- Complete VETC phenotype: Diffuse VETC images (i.e., « VETC-HCC », ≥55% of tumor surface)
Tumoral differentiation (WHO)	- I : Well differentiated<br>- II : Moderately differentiated<br>- III : Poorly differentiated
Tumoral necrosis	Percentage of tumoral necrosis (0 to 100).
Tumoral fibrosis	Percentage of tumoral fibrosis (0 to 100).
Tumoral inflammatory infiltrate	Semi-quantitative (Absent, Low, Moderate, High).
Tumoral steatosis	Percentage of steatotic tumoral cells (0 to 100).
Tumoral cholestasis	Absent / Present

Table S2. Correlation between histopathological criteria and response in the discovery cohort

**Characteristic**	**No response** N = 122[1]	**Response** N = 86[1]	**p-value**[2]
Macrotrabecular massive-HCC	39 (32%)	19 (22%)	0.12
Steatohepatitic-HCC	15 (12%)	9 (10%)	0.7
Squirrhous-HCC	10 (8.2%)	6 (7.0%)	0.7
Clear cell-HCC	7 (5.7%)	2 (2.3%)	0.3
Not otherwise specified-HCC	50 (41%)	48 (56%)	**0.035**
Tumoral necrosis	33 (27%)	25 (29%)	0.7
Tumoral inflammatory infiltrate			0.5
Absent	55 (45%)	36 (42%)	
Low	35 (29%)	28 (33%)	
Intermediate	22 (18%)	11 (13%)	
High	10 (8.2%)	11 (13%)	
Tumoral steatosis	29 (24%)	23 (27%)	0.6
Tumoral cholestasis	10 (8.2%)	10 (12%)	0.4
Differentiation (WHO)			0.7
I	44 (36%)	36 (42%)	
II	62 (51%)	40 (47%)	
III	16 (13%)	10 (12%)	
VETC phenotype			**0.002**
Non or Incomplete VETC-HCC	111 (91%)	65 (76%)	
VETC-HCC	11 (9.0%)	21 (24%)	

[1]n (%); [2]Pearson's Chi-squared test

Table S3. Contingency table between HES and CD34 assessment for the VETC phenotype in the discovery cohort

	No VETC (CD34)	Incomplete VETC-HCC (CD34)	VETC-HCC (CD34)	Total
No VETC (HES)	115	5	1	121
Incomplete VETC-HCC (HES)	1	18	3	22
VETC-HCC (HES)	0	0	19	19
Total	116	23	23	162

Table S4. Agreement between CD34 and HES staining for VETC phenotype in the discovery cohort

**Agreement metric**	**Kappa**	**Lower 95% CI**	**Upper 95% CI**	**ASE**	**z-score**	**p-value**
Unweighted kappa	0.856	0.772	0.940	0.043	19.907	<0.001
Weighted kappa (linear)	0.890	0.821	0.959	0.035	25.429	<0.001

Table S5. Characteristics of HCC with VETC images in the discovery cohort

Characteristic	Non-VETC-HCC N = 151[1]	Incomplete and Complete VETC-HCC N = 57[1]	p-value[2]
Viral hepatitis (HBV/HCV)	66 (44%)	27 (47%)	0.6
Child Pugh class			0.2
No cirrhosis	50 (33%)	26 (46%)	
A	82 (54%)	23 (40%)	
B	19 (13%)	8 (14%)	
Macrovascular invasion	65 (43%)	22 (39%)	0.6
Extrahepatic spread	53 (35%)	21 (37%)	0.8
AFP[3]	49.5 (6.0-5,077.0)	133.0 (6.4-3,826.0)	0.8
CRP[4]	11.5 (4.0-31.0)	11.0 (6.0-60.0)	0.8
CRAFITY[5]			0.5
low	35 (29%)	13 (29%)	
intermediate	51 (42%)	15 (33%)	
high	35 (29%)	17 (38%)	
Macrotrabecular massive-HCC	37 (25%)	21 (37%)	0.077
Steatohepatitic-HCC	23 (15%)	1 (1.8%)	0.007
Squirrhous-HCC	15 (9.9%)	1 (1.8%)	0.076
Clear cell-HCC	7 (4.6%)	2 (3.5%)	0.9
Not otherwise specified-HCC	66 (44%)	32 (56%)	0.11
p53 expression[6]	25 (34%)	9 (28%)	0.6
PD-L1 > 0 (TPS and/or CPS)[7]	24 (42%)	9 (31%)	0.3
B-catenin expression[8]	14 (17%)	14 (41%)	0.005
CD8+ T cells (/mm²)[6]	74.2 (29.2-237.4)	45.6 (27.9-128.7)	0.066
CD3+ T cells (/mm²)[9]	285.5 (112.0-557.0)	157.1 (92.9-335.4)	0.040
Objective Response Rate			0.086

Response	57 (38%)	29 (51%)

[1]Median (Q1 - Q3); n (%);[2]Wilcoxon rank sum test; Pearson's Chi-squared test [3]Available for 205 cases; [4]Available for 169 cases; [5]Available for 166 cases; [6]Available for 106 cases; [7]Available for 86 cases; [8]Available for 117 cases; [9]Available for 100 cases

Table S6. Sensitivity Analysis of VETC Thresholds in patients treated with atezo+bev using PFS (Discovery Cohort)

Threshold (%)	HR	Lower 95% CI	Upper 95% CI	p-value	n patients > threshold	n patients < threshold	Proportion patients > threshold
**55**	**0.528**	**0.336**	**0.828**	**0.005**	**32**	**176**	**0.154**
**50**	**0.618**	**0.411**	**0.929**	**0.021**	**38**	**170**	**0.183**
**60**	**0.615**	**0.389**	**0.972**	**0.037**	**29**	**179**	**0.139**
40	0.710	0.491	1.026	0.068	49	159	0.236
45	0.716	0.486	1.054	0.090	42	166	0.202
65	0.742	0.455	1.208	0.230	24	184	0.115
35	0.811	0.571	1.152	0.242	54	154	0.260
70	0.780	0.466	1.306	0.345	21	187	0.101
90	0.705	0.331	1.503	0.365	10	198	0.048
30	0.869	0.619	1.221	0.418	58	150	0.279
75	0.821	0.456	1.476	0.509	16	192	0.077
15	0.901	0.658	1.234	0.516	72	136	0.346
25	0.924	0.667	1.280	0.635	64	144	0.308
85	0.873	0.461	1.653	0.676	13	195	0.062
20	0.940	0.683	1.292	0.701	69	139	0.332
10	0.963	0.708	1.311	0.810	78	130	0.375
5	0.964	0.710	1.309	0.816	80	128	0.385
80	0.932	0.506	1.718	0.822	14	194	0.067
95	0.967	0.308	3.034	0.954	4	204	0.019

Table S7. Clinical and biological characteristics of *TP53* and *CTNNB1* mutated HCC in patients from Beaujon hospital in the discovery cohort

**Characteristic**	***CTNNB1*-mutated expression** N = 25[1]	***TP53*-mutated expression** N = 31[1]	**Double positive** N = 3[1]	**Double negative** N = 46[1]	**p-value**[2]
AFP (ng/mL)[3]	38.0 (4.0-2,542.5)	849.0 (45.0-14,300.0)	11.0 (4.0-316,831.0)	61.0 (5.4-5,215.0)	0.4
CRAFITY score[4]					0.3
low	6 (26%)	3 (10%)	0 (0%)	13 (31%)	
intermediate	11 (48%)	12 (41%)	2 (67%)	14 (33%)	
high	6 (26%)	14 (48%)	1 (33%)	15 (36%)	
Viral etiology	9 (36%)	18 (58%)	3 (100%)	20 (43%)	0.10
Differentiation (WHO)					<0.001
I	15 (60%)	2 (6.5%)	0 (0%)	17 (37%)	
II	9 (36%)	18 (58%)	3 (100%)	23 (50%)	
III	1 (4.0%)	11 (35%)	0 (0%)	6 (13%)	
BCLC stage					0.2
B	11 (44%)	6 (19%)	1 (33%)	12 (26%)	
C	14 (56%)	25 (81%)	2 (67%)	34 (74%)	
Histological subtype					
NOS	22 (88%)	7 (23%)	0 (0%)	22 (48%)	
CC	0 (0%)	2 (6.5%)	0 (0%)	1 (2.2%)	
MTM	2 (8.0%)	17 (55%)	3 (100%)	9 (20%)	
SH	1 (4.0%)	1 (3.2%)	0 (0%)	7 (15%)	
SQ	0 (0%)	4 (13%)	0 (0%)	7 (15%)	
VETC					0.6
Non or Incomplete VETC-HCC	21 (84%)	28 (90%)	2 (67%)	40 (87%)	
VETC-HCC	4 (16%)	3 (9.7%)	1 (33%)	6 (13%)	
CD8+ T cells density[5]	38.4 (22.2-92.1)	136.2 (42.4-371.1)	5.3 (0.0-52.4)	61.9 (33.6-141.7)	0.013

Proliferation index (Ki67, %)[6]	5.0 (3.6-7.8)	28.7 (19.6-34.9)	25.0 (5.0-33.3)	10.7 (4.1-16.5)	<0.001
TPS > 1%[7]					0.2
0	15 (94%)	17 (68%)	3 (100%)	29 (83%)	
1	1 (6.3%)	8 (32%)	0 (0%)	6 (17%)	
Objective Response Rate					0.9
No response	16 (64%)	19 (61%)	2 (67%)	27 (59%)	
Response	9 (36%)	12 (39%)	1 (33%)	19 (41%)	

[1]Median (IQR); n (%); [2]Kruskal-Wallis rank sum test; Fisher's exact test; [3]Available for 205 cases; [4]Available for 164 cases; [5]Available for 106 cases; [6]Available for 95 cases; [7]Available for 86 cases

Table S8. Univariable and multivariable Cox regression analyses of prognostic factors for progression-free and overall survival in the discovery cohort.

	Univariable (PFS)				Multivariable (PFS)			Univariable (OS)				Multivariable (OS)		
**Characteristic**	**N**	**HR**	**95% CI**	**p-value**	**HR**	**95% CI**	**p-value**	**N**	**HR**	**95% CI**	**p-value**	**HR**	**95% CI**	**p-value**
Sex	208							208						
Male		—	—		—	—			—	—		—	—	
Female		0.82	0.52, 1.28	0.4	0.78	0.49, 1.25	0.3		0.98	0.59, 1.61	0.9	0.95	0.57, 1.61	0.9
Age	208							208						
<65 years		—	—		—	—			—	—		—	—	
>65 years		0.96	0.70, 1.32	0.8	1.11	0.75, 1.63	0.6		0.95	0.66, 1.37	0.8	1.06	0.70, 1.63	0.8
ECOG PS	208							208						
0		—	—		—	—			—	—		—	—	
≥ 1		1.33	0.98, 1.79	0.064	1.37	0.98, 1.93	0.066		1.74	1.23, 2.46	0.002	1.69	1.14, 2.50	0.008
Child Pugh	208							208						
No cirrhosis		—	—		—	—			—	—		—	—	
A		0.77	0.56, 1.06	0.10	0.74	0.52, 1.05	0.089		0.67	0.46, 0.98	0.037	0.72	0.48, 1.06	0.10
B		1.56	0.98, 2.47	0.061	1.80	1.04, 3.12	0.035		2.28	1.38, 3.79	0.001	2.84	1.54, 5.22	<0.001
Viral etiology	208							208						
Yes		—	—		—	—			—	—		—	—	
No		1.13	0.84, 1.52	0.4	1.30	0.90, 1.87	0.2		1.22	0.87, 1.73	0.3	1.45	0.97, 2.18	0.071
BCLC	208							208						
B		—	—		—	—			—	—		—	—	
C		1.01	0.74, 1.38	>0.9	1.09	0.77, 1.54	0.6		1.04	0.73, 1.49	0.8	1.05	0.70, 1.58	0.8
AFP > 400 ng.mL^{-1}	205							205						
No		—	—		—	—			—	—		—	—	
Yes		1.56	1.15, 2.11	0.004	1.78	1.28, 2.46	<0.001		1.45	1.02, 2.05	0.036	1.78	1.22, 2.59	0.003
Previous treatment	208							208						
No		—	—		—	—			—	—		—	—	
Yes		0.88	0.62, 1.25	0.5	0.98	0.67, 1.42	0.9		0.75	0.49, 1.14	0.2	0.87	0.56, 1.37	0.6
Center	208							208						
Beaujon		—	—						—	—				
Other		0.82	0.59, 1.14	0.2					0.86	0.59, 1.25	0.4			

Histological subtype	208							208						
NOS		—	—		—	—			—	—		—	—	
CC		1.82	0.91, 3.63	0.090	2.26	1.07, 4.77	0.032		1.63	0.78, 3.42	0.2	1.68	0.76, 3.70	0.2
MTM		1.35	0.95, 1.92	0.10	1.37	0.94, 2.00	0.10		1.36	0.90, 2.05	0.15	1.32	0.85, 2.06	0.2
SH		1.07	0.66, 1.72	0.8	1.07	0.55, 2.06	0.8		1.28	0.74, 2.24	0.4	1.51	0.68, 3.35	0.3
SQ		1.77	1.04, 3.04	0.037	1.57	0.90, 2.76	0.11		1.87	1.02, 3.42	0.041	1.60	0.84, 3.05	0.15
VETC	208							208						
Non- or Incomplete-VETC-HCC		—	—		—	—			—	—		—	—	
VETC-HCC		0.53	0.34, 0.83	0.005	0.42	0.26, 0.68	<0.001		0.53	0.31, 0.91	0.022	0.46	0.26, 0.82	0.009
Differentiation	208							208						
Well		—	—						—	—				
Moderately		1.09	0.79, 1.49	0.6					1.12	0.78, 1.62	0.5			
Poorly		1.17	0.72, 1.89	0.5					1.10	0.64, 1.88	0.7			
Inflammatory infiltrate	208							208						
Absent		—	—						—	—				
Low		0.96	0.68, 1.36	0.8					1.08	0.72, 1.62	0.7			
Intermediate		0.89	0.58, 1.37	0.6					1.25	0.78, 2.03	0.4			
High		1.09	0.66, 1.83	0.7					0.99	0.53, 1.85	>0.9			
Tumoral necrosis	208							208						
No		—	—						—	—				
Yes		1.20	0.86, 1.66	0.3					1.05	0.72, 1.54	0.8			
Tumoral steatosis	208							208						
No		—	—		—	—			—	—		—	—	
Yes		0.76	0.54, 1.07	0.11	0.80	0.50, 1.28	0.4		0.70	0.47, 1.05	0.081	0.61	0.34, 1.09	0.10
Tumoral cholestasis	208							208						
No		—	—						—	—				
Yes		0.84	0.50, 1.41	0.5					1.07	0.61, 1.91	0.8			

Abbreviations: CI = Confidence Interval, HR = Hazard Ratio

Table S9. Progression-Free and Overall Survival Cox model including treatment interaction (VETC x treatment) in the validation cohort.

	PFS			OS		
**Characteristic**	**HR**	**95% CI**	**p-value**	**HR**	**95% CI**	**p-value**
AFP > 400 ng/mL vs AFP < 400 ng/mL	1.17	0.76, 1.79	0.5	1.47	0.93, 2.32	0.10
ECOG PS ≥ 1 vs 0	1.28	0.84, 1.95	0.3	1.66	1.02, 2.68	0.040
BCLC C vs B	1.19	0.75, 1.90	0.5	1.66	0.98, 2.81	0.058
Age ≥ 65 vs <65 years old	0.71	0.47, 1.07	0.10	0.66	0.40, 1.07	0.088
Female vs Male	1.14	0.68, 1.93	0.6	0.94	0.51, 1.76	0.9
Previous locoregional treatment vs none	1.21	0.74, 1.96	0.5	1.36	0.81, 2.29	0.2
Child Pugh class						
No cirrhosis	—	—		—	—	
A	0.70	0.45, 1.10	0.13	1.04	0.63, 1.71	0.9
B	0.96	0.45, 2.02	0.9	1.52	0.68, 3.38	0.3
Etiology : Non-viral vs viral	1.20	0.76, 1.88	0.4	1.80	1.09, 2.95	0.021
**Interaction term : VETC status X treatment**	0.28	0.10, 0.79	0.016	0.15	0.05, 0.48	0.001
**Adjusted subgroup hazard ratios**						
VETC-HCC vs non-VETC-HCC in patients treated with durva+treme	2.44	1.12–5.29	0.024	3.90	1.66–9.15	0.002
VETC-HCC vs non-VETC-HCC in patients treated with atezo+bev	0.69	0.35–1.36	0.280	0.61	0.29–1.3	0.2
Atezo+bev vs durva+treme in patients with VETC-HCC	0.43	0.17–1.06	0.067	0.19	0.07–0.51	0.001
Atezo+bev vs durva+treme in	1.52	0.93–2.48	0.095	1.22	0.69–2.13	0.5

patients with non-VETC-HCC	

Abbreviations: CI = Confidence Interval, HR = Hazard Ratio

Table S10. Progression-Free and Overall Survival Cox model including treatment interaction (VETC x treatment) in the whole cohort (discovery + validation).

	PFS			OS		
**Characteristic**	**HR**	**95% CI**	**p-value**	**HR**	**95% CI**	**p-value**
AFP ≥ 400 ng/mL vs AFP < 400 ng/mL	1.47	1.15, 1.88	0.002	1.56	1.18, 2.06	0.002
ECOG PS ≥ 1 vs 0	1.32	1.03, 1.69	0.029	1.76	1.33, 2.33	<0.001
BCLC C vs B	1.14	0.87, 1.48	0.3	1.26	0.93, 1.70	0.13
Age ≥ 65 vs <65 years old	0.89	0.67, 1.17	0.4	0.86	0.63, 1.18	0.3
Female vs Male	0.95	0.68, 1.32	0.7	0.97	0.66, 1.42	0.9
Previous locoregional treatment vs none	1.16	0.89, 1.53	0.3	1.22	0.90, 1.67	0.2
Child Pugh Score						
No cirrhosis	—	—		—	—	
A	0.71	0.54, 0.92	0.011	0.76	0.57, 1.03	0.081
B	1.25	0.83, 1.88	0.3	1.94	1.23, 3.04	0.004
Etiology : Non-viral vs viral	1.17	0.89, 1.54	0.3	1.46	1.07, 1.98	0.016
**Interaction term : VETC status X treatment**	0.22	0.10, 0.51	<0.001	0.15	0.06, 0.38	<0.001
**Adjusted subgroup hazard ratios**						
VETC-HCC vs non-VETC-HCC in patients treated with durva+treme	2.50	1.18–5.29	0.017	3.54	1.56–8.03	0.002
VETC-HCC vs non-VETC-HCC in patients treated with atezo+bev	0.56	0.38–0.81	0.002	0.54	0.35–0.83	0.005
Atezo+bev vs durva+treme in patients with VETC-HCC	0.34	0.16–0.69	0.003	0.19	0.09–0.42	<0.001

Atezo+bev vs durva+treme in patients with non-VETC-HCC	1.51	0.98–2.31	0.06	1.26	0.77–2.07	0.3

Abbreviations: CI = Confidence Interval, HR = Hazard Ratio

Table S11. Sensitivity analyses of overall survival restricted to patients with OS ≥ 3 months.

Model	Term	HR	Lower 95% CI	Upper 95% CI	p-value
Discovery cohort (patients treated with Atezo+Bev) – OS ≥ 3 months	VETC-HCC (Yes vs No)	0.576	0.362	0.915	0.020
Discovery cohort (patients treated with Atezo+Bev) – OS ≥ 3 months	AFP > 400 ng/mL	1.660	1.201	2.295	0.002
Discovery cohort (patients treated with Atezo+Bev) – OS ≥ 3 months	Age > 65 years	1.131	0.773	1.654	0.526
Discovery cohort (patients treated with Atezo+Bev) – OS ≥ 3 months	BCLC C vs B	1.086	0.770	1.531	0.637
Discovery cohort (patients treated with Atezo+Bev) – OS ≥ 3 months	Child-Pugh A vs no cirrhosis	0.795	0.565	1.119	0.189
Discovery cohort (patients treated with Atezo+Bev) – OS ≥ 3 months	Child-Pugh B vs no cirrhosis	2.059	1.164	3.643	0.013
Discovery cohort (patients treated with Atezo+Bev) – OS ≥ 3 months	ECOG PS ≥ 1	1.601	1.152	2.227	0.005
Discovery cohort (patients treated with Atezo+Bev) – OS ≥ 3 months	Female vs male	0.902	0.575	1.414	0.653
Discovery cohort (patients treated with Atezo+Bev) – OS ≥ 3 months	Non-viral vs viral etiology	1.489	1.040	2.132	0.030
Discovery cohort (patients treated with Atezo+Bev) – OS ≥ 3 months	Previous locoregional treatment	1.312	0.923	1.867	0.131
Validation cohort – OS ≥ 3 months	VETC-HCC (Yes vs No) in patients treated with durva+treme	3.232	0.988	10.570	0.052
Validation cohort – OS ≥ 3 months	Interaction: VETC status x treatment	0.254	0.064	1.001	0.050

Validation cohort – OS ≥ 3 months	AFP > 400 ng/mL	1.383	0.827	2.312	0.217
Validation cohort – OS ≥ 3 months	Age > 65 years	0.875	0.497	1.540	0.643
Validation cohort – OS ≥ 3 months	BCLC C vs B	2.084	1.141	3.808	0.017
Validation cohort – OS ≥ 3 months	Child-Pugh A vs no cirrhosis	1.124	0.638	1.981	0.686
Validation cohort – OS ≥ 3 months	Child-Pugh B vs no cirrhosis	1.901	0.766	4.722	0.166
Validation cohort – OS ≥ 3 months	ECOG PS ≥ 1	1.249	0.740	2.106	0.405
Validation cohort – OS ≥ 3 months	Female vs male	0.692	0.335	1.429	0.320
Validation cohort – OS ≥ 3 months	Non-viral vs viral etiology	1.882	1.067	3.321	0.029
Validation cohort – OS ≥ 3 months	Previous locoregional treatment	1.498	0.839	2.673	0.172
Validation cohort – OS ≥ 3 months	Treatment: atezo+bev vs durva+treme	1.572	0.807	3.061	0.184
Whole cohort – OS ≥ 3 months	VETC-HCC (Yes vs No) in patients treated with durva+treme	3.310	1.045	10.488	0.042
Whole cohort – OS ≥ 3 months	Interaction: VETC status × treatment	0.174	0.051	0.598	0.005
Whole cohort – OS ≥ 3 months	AFP > 400 ng/mL	1.586	1.163	2.162	0.004
Whole cohort – OS ≥ 3 months	Age > 65 years	1.065	0.742	1.526	0.734
Whole cohort – OS ≥ 3 months	BCLC C vs B	1.154	0.832	1.601	0.389
Whole cohort – OS ≥ 3 months	Child-Pugh A vs no cirrhosis	0.801	0.576	1.114	0.188
Whole cohort – OS ≥ 3 months	Child-Pugh B vs no cirrhosis	2.080	1.211	3.571	0.008
Whole cohort – OS ≥ 3 months	ECOG PS ≥ 1	1.610	1.178	2.201	0.003

Whole cohort – OS ≥ 3 months	Female vs male	0.936	0.612	1.431	0.760
Whole cohort – OS ≥ 3 months	Non-viral vs viral etiology	1.467	1.042	2.064	0.028
Whole cohort – OS ≥ 3 months	Previous locoregional treatment	1.372	0.983	1.915	0.063
Whole cohort – OS ≥ 3 months	Treatment: atezo+bev vs durva+treme	1.530	0.842	2.782	0.163

Fig. S1. Flowchart.

A : Discovery Cohort

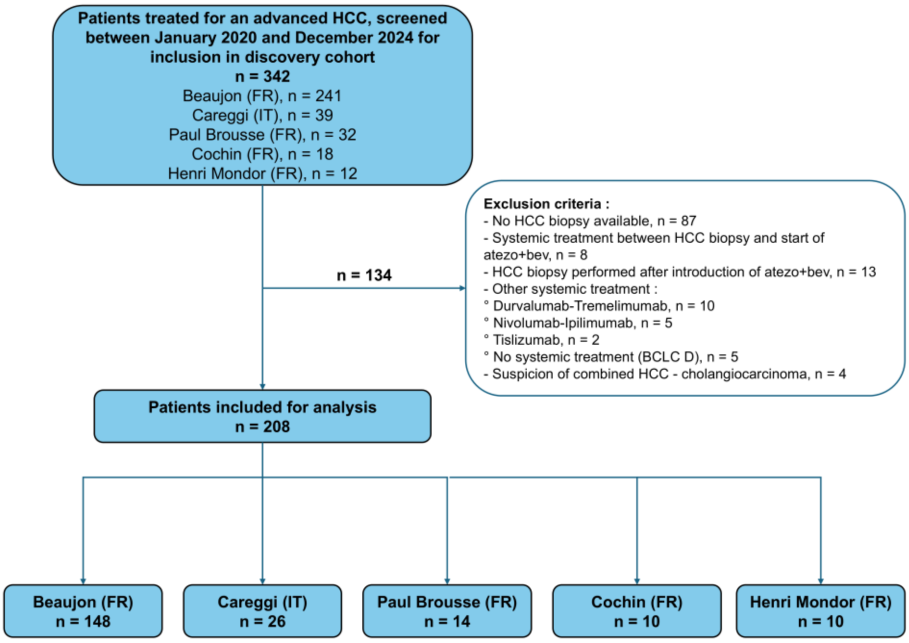

B : Validation cohort

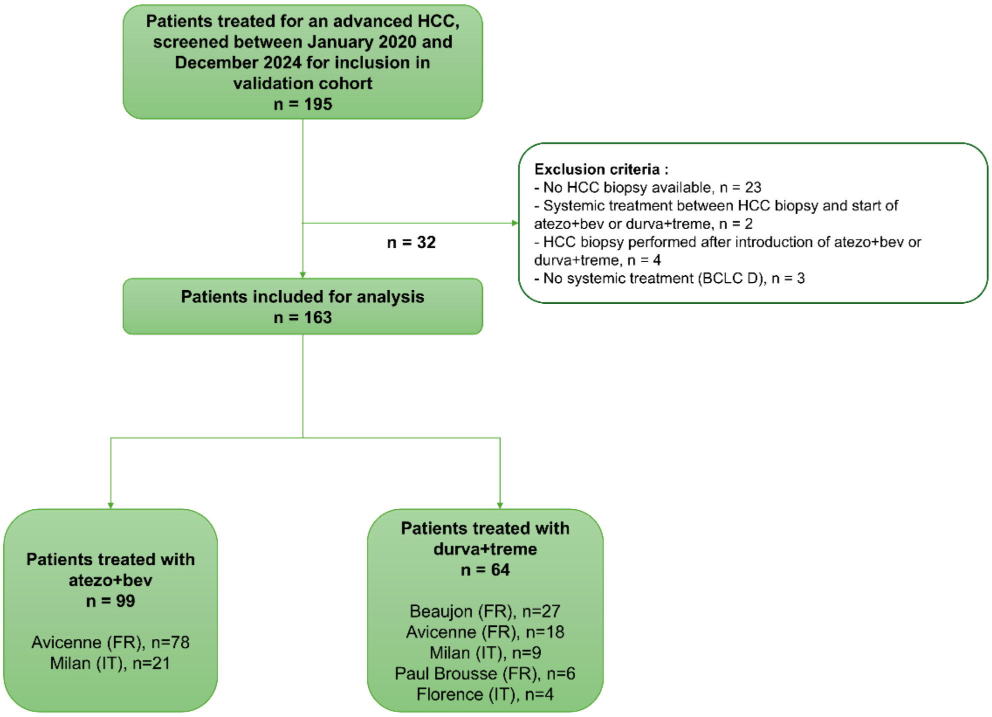

Fig. S2. Survival outcomes according to serum AFP and CRAFITY score in the discovery cohort

A, B: Progression-Free Survival and Overall Survival according to serum AFP (< 400 / > 400 ng.mL^{-1}). **C, D:** Progression-Free Survival and Overall Survival according to CRAFITY score (Low, Intermediate, High). HRs from univariate Cox regression are displayed with their 95% CI and p-value (Wald test).

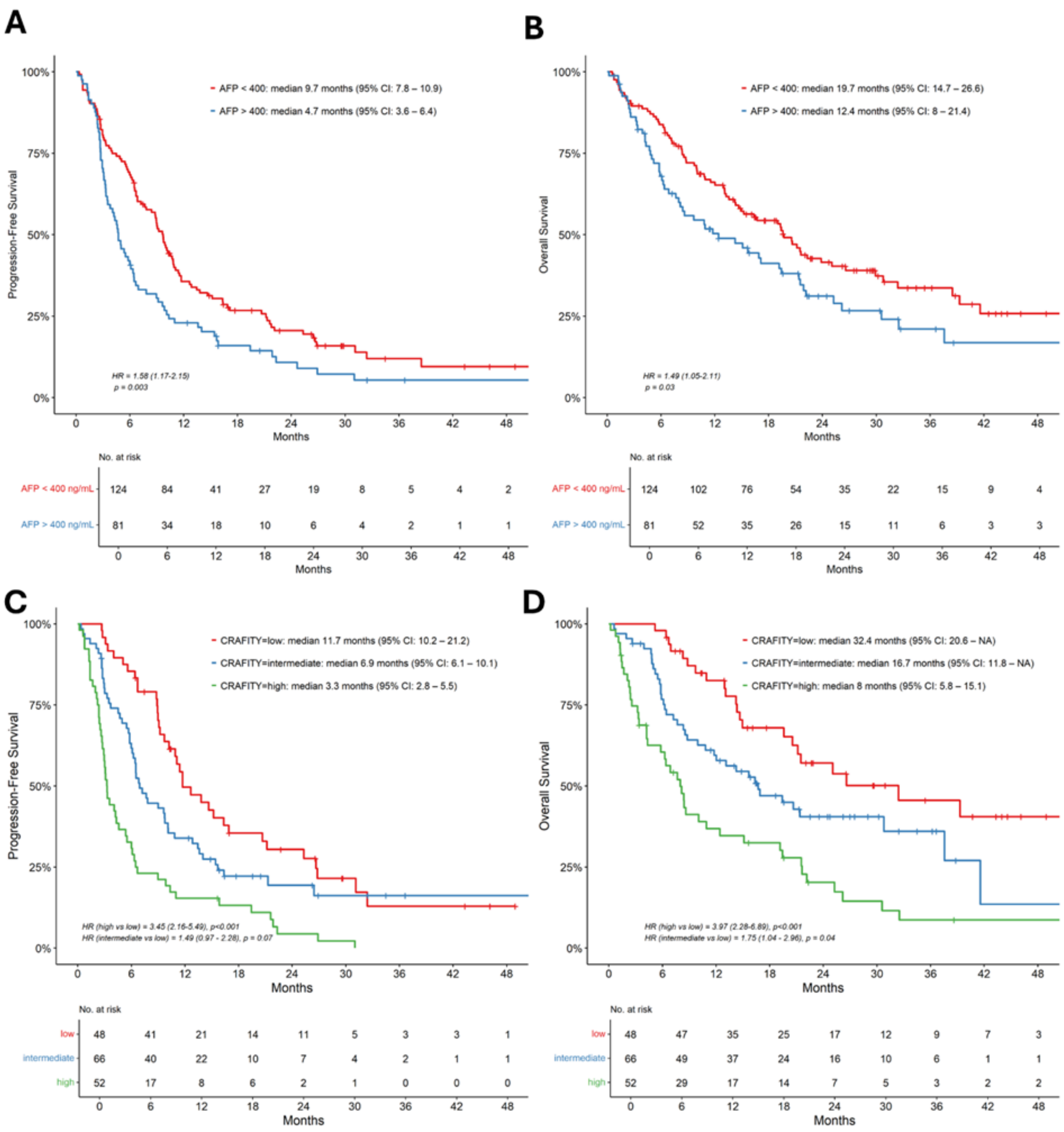

Fig. S3. Overall survival according to best response per mRECIST in the discovery cohort.

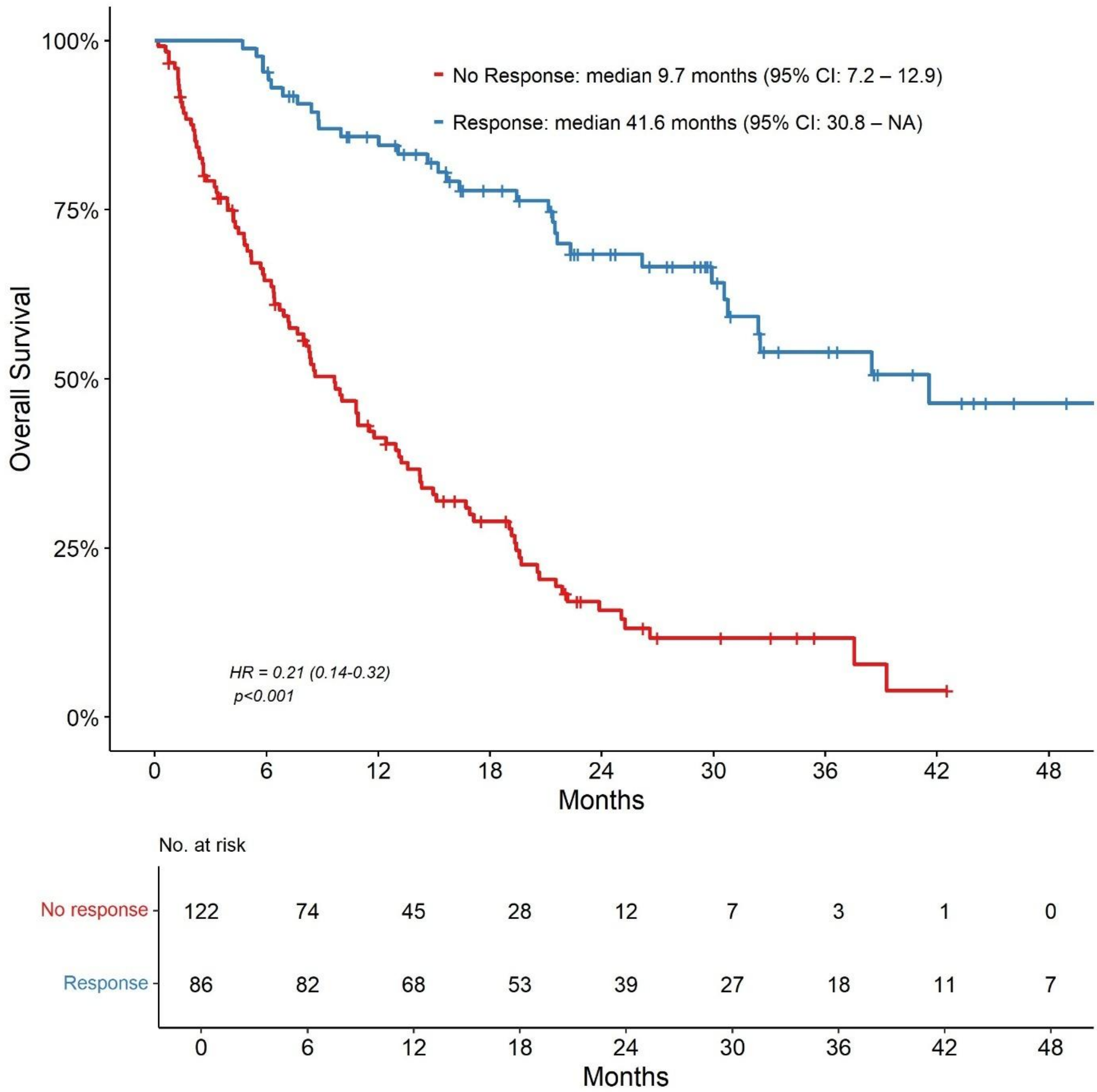

Fig. S4. Survival outcomes in SQ, CC and SH-HCC in the discovery cohort

A, B: Progression-Free Survival and Overall Survival in SQ-HCC and NOS-HCC
C, D: Progression-Free Survival and Overall Survival in CC-HCC and NOS-HCC
E, F: Progression-Free Survival and Overall Survival in SH-HCC and NOS-HCC

HRs from univariate Cox regression are displayed with their 95% CI and p-value (Wald test).

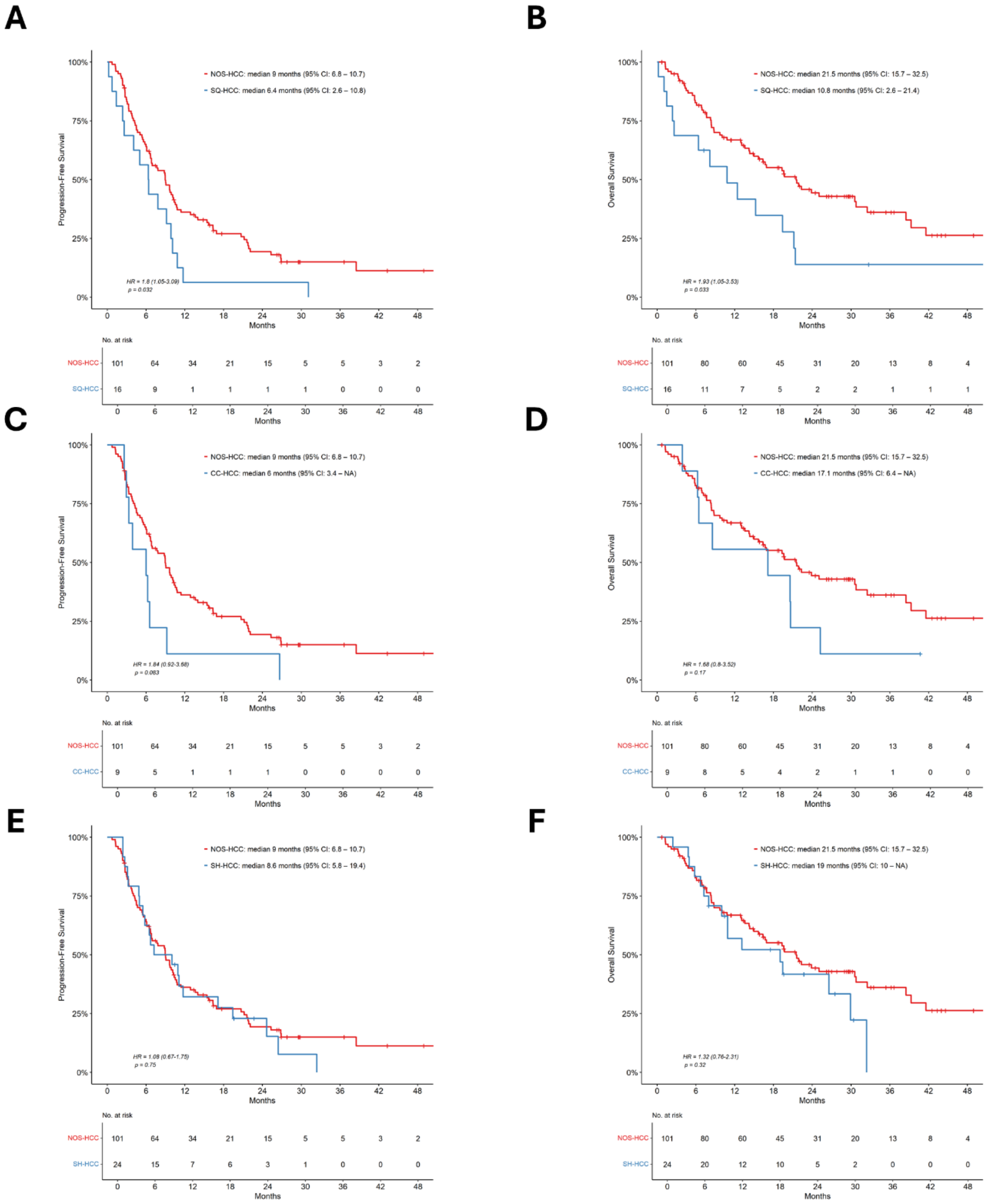

Fig. S5. Representative histological images of the 9 complete VETC-HCC cases diagnosed on HES only from the discovery cohort

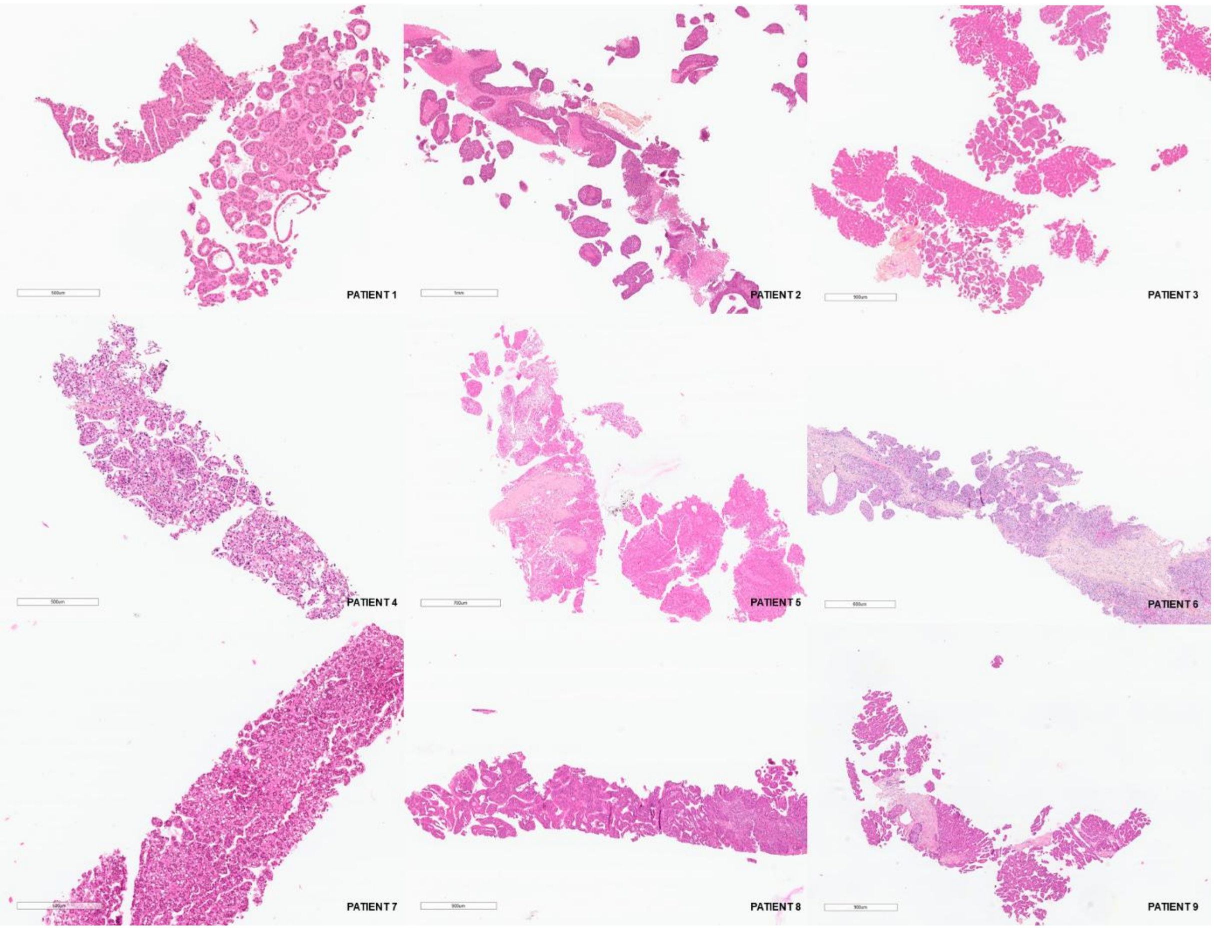

Fig. S6. Survival outcomes in incomplete-VETC-HCC, compared to complete VETC-HCC and non-VETC-HCC in the discovery cohort.

A, B: Progression-Free Survival and Overall Survival in complete VETC-HCC, incomplete-VETC-HCC and non-VETC-HCC.

C, D: Progression-Free Survival and Overall Survival in subgroup of patients who had available CD34 immunostaining, according to VETC phenotype. HRs from univariate Cox regression are displayed with their 95% CI and p-value (Wald test).

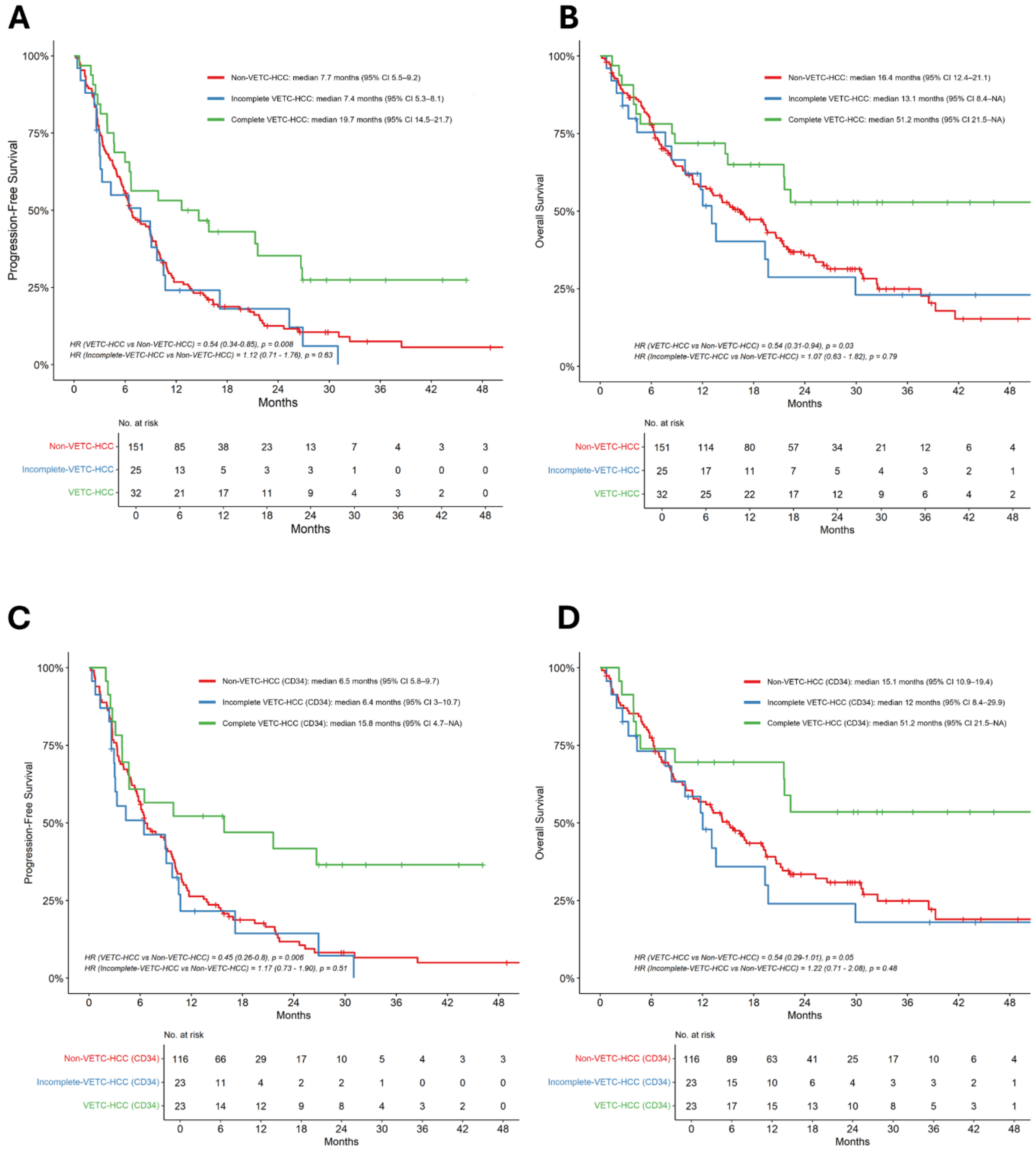

Fig. S7. Effect of VETC threshold on Hazard ratio for Progression-Free survival

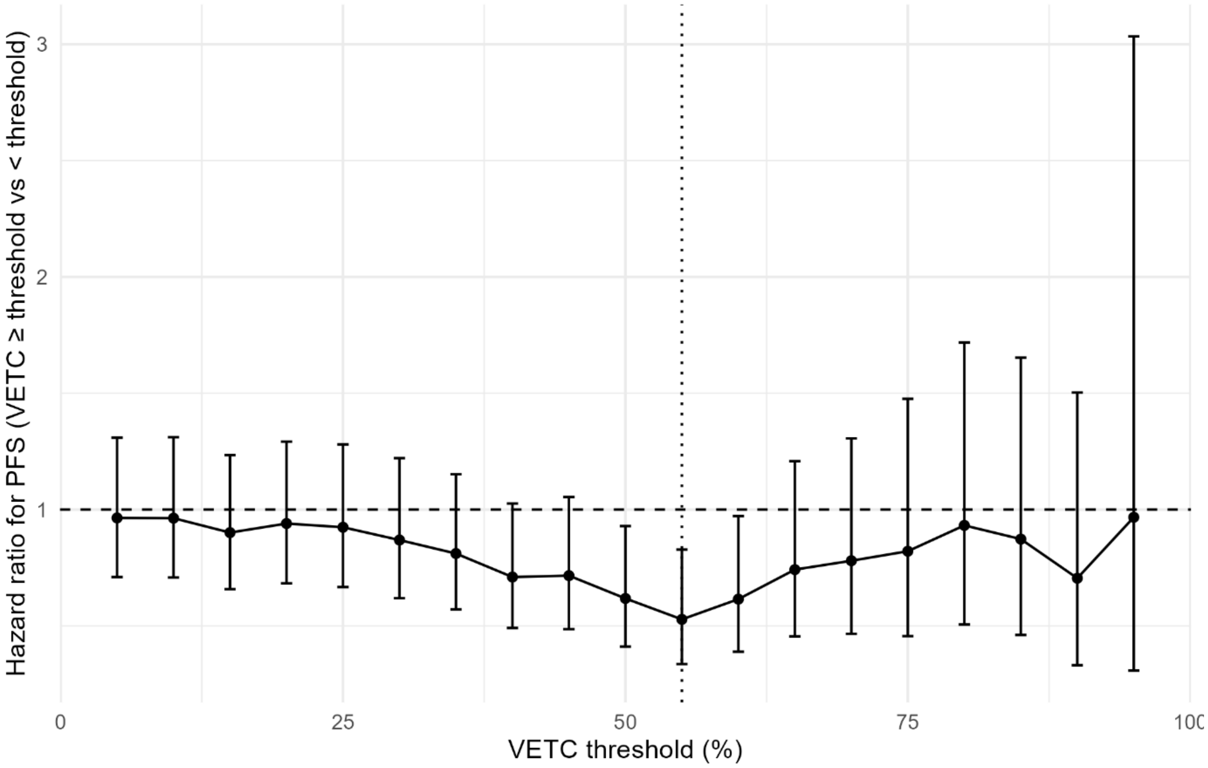

Horizontal dashed line: Hazard Ratio = 1. Vertical dotted line: 55% cut-off for VETC.

Fig. S8. Survival outcomes according to CD8 infiltrate, *TP53* and *CTNNB1* status in patients from Beaujon Hospital in the discovery cohort.

A, B: Progression-Free Survival and Overall Survival in HCC with high or low-CD8 infiltrate

C, D: Progression-Free Survival and Overall Survival according to p53 expression.

E, F: Progression-Free Survival and Overall Survival according to GS/β-catenin expression. HRs from univariate Cox regression are displayed with their 95% CI and p-value (Wald test).

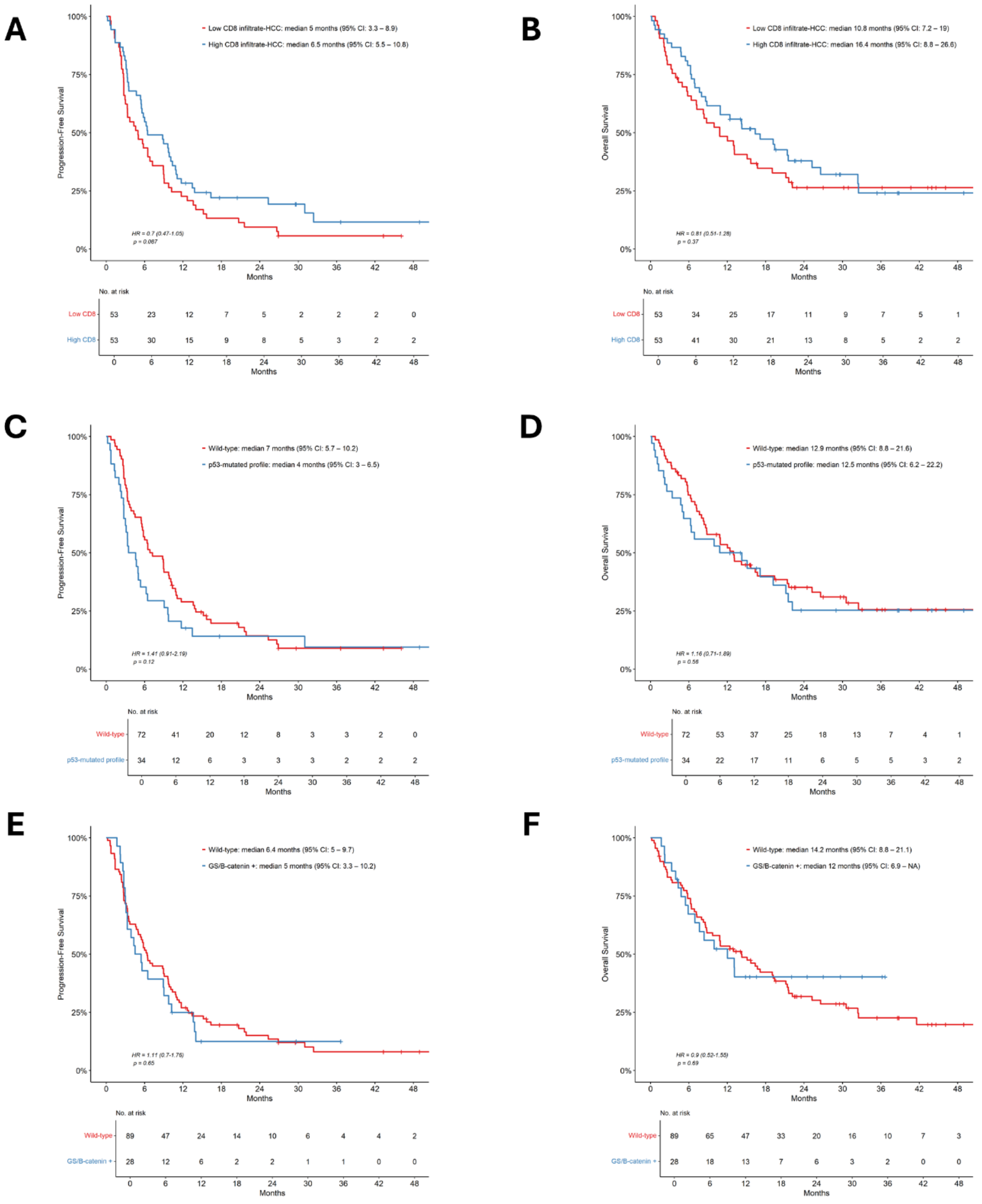

Fig. S9. Predictive impact of atezo+bev on survival of patients with complete VETC HCC (≥55%) in the whole cohort (discovery+validation)

A: Progression-Free Survival according to atezo+bev (red) or durva-treme (blue) treatments in complete VETC-HCC. B: Progression-Free Survival according to atezo+bev (red) or durva-treme (blue) treatments in non or incomplete-VETC-HCC. C: Overall Survival according to atezo+bev (red) or durva-treme (blue) treatments in complete VETC-HCC. D: Overall Survival according to atezo+bev (red) or durva-treme (blue) treatments in non or incomplete-VETC-HCC. HRs from univariate Cox regression are displayed with their 95% CI and p-value (Wald test).

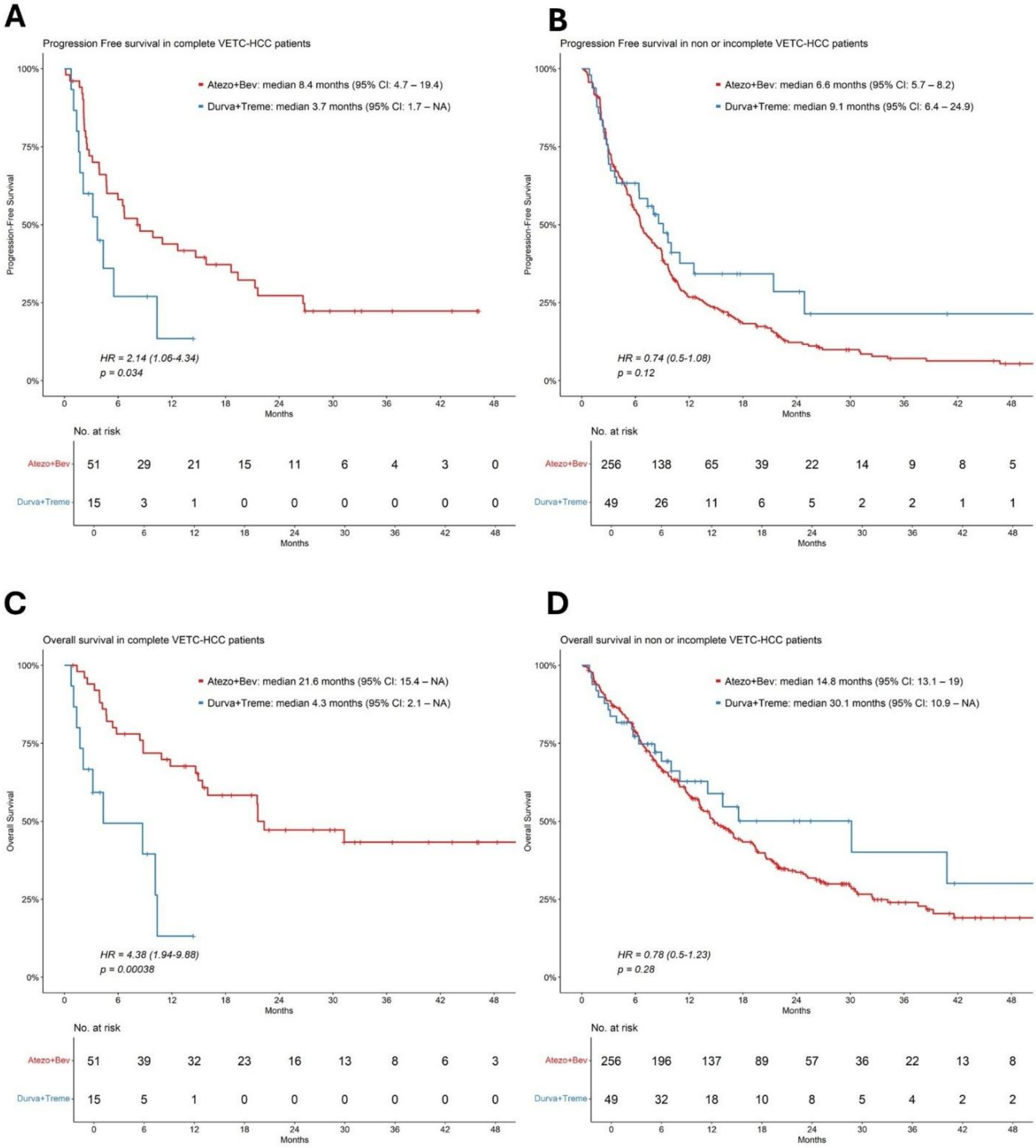

Supplement: Multimedia component 1 [file mmc1.pdf]
