# Supplementary material for: Vessels encapsulating tumor clusters predict better outcomes in advanced hepatocellular carcinoma treated with atezolizumab–bevacizumab
Source: JHEP Rep. 2026 Apr 29;8(7):101874. doi: 10.1016/j.jhepr.2026.101874 (PMC13276544; doi:10.1016/j.jhepr.2026.101874)
Supplement: Multimedia component 2 [file mmc2.pdf]

JHEP|Reports

JHEP Reports

CTAT methods

Tables for a "Complete, Transparent, Accurate and Timely account" (CTAT) are now mandatory for all revised submissions. The aim is to enhance the reproducibility of methods.

- Only include the parts relevant to your study
- Refer to the CTAT in the main text as 'Supplementary CTAT Table'
- Do not add subheadings
- Add as many rows as needed to include all information
- Only include one item per row

If the CTAT form is not relevant to your study, please outline the reasons why:

1.1 Antibodies

Name	Citation	Supplier	Cat no.	Clone no.
CD3		Dako	A045229-2	A0452
CD8		Dako	M7103	C8/144B
PD-L1 (QR1)		Quartett	1-PR292-02	QR1
Glutamine synthetase		Chemicon	MAB302	GS-6
β-catenin		BD Biosciences	610153	14/Beta-Catenin
p53		Dako	M700101	DO7

1.2 Cell lines

Name	Citation	Supplier	Cat no.	Passage no.	Authentication test method

1.3 Organisms

Name	Citation	Supplier	Strain	Sex	Age	Overall n number

1.4 Sequence based reagents

Name	Sequence	Supplier

1.5 Biological samples

Description	Source	Identifier

1.6 Deposited data

Name of repository	Identifier	Link

1.7 Software

Software name	Manufacturer	Version
R software	R foundation	v4.3.3

1.8 Other (*e.g.* drugs, proteins, vectors etc.)

1.9 Please provide the details of the corresponding methods author for the manuscript:

Aurélie Beaufrère, MD, PhD
Department of Pathology, Beaujon Hospital,100 boulevard du Général Leclerc, Clichy, 92110, France
aurelie.beaufrere@aphp.fr

2.0 Please confirm for randomised controlled trials all versions of the clinical protocol are included in the submission. These will be published online as supplementary information.
