# Supplementary material for: Vessels encapsulating tumor clusters predict better outcomes in advanced hepatocellular carcinoma treated with atezolizumab–bevacizumab
Source: JHEP Rep. 2026 Apr 29;8(7):101874. doi: 10.1016/j.jhepr.2026.101874 (PMC13276544; doi:10.1016/j.jhepr.2026.101874)

Research article

JHEP|Reports

Vessels encapsulating tumor clusters predict better outcomes in advanced hepatocellular carcinoma treated with atezolizumab–bevacizumab

Authors

Alexandre Sayadi, Claudia Campani, Astrid Laurent-Bellue, …, Mohamed Bouattour, Valérie Paradis, Aurélie Beaufrère

Correspondence

aurelie.beaufrere@aphp.fr (A. Beaufrère).

Graphical abstract

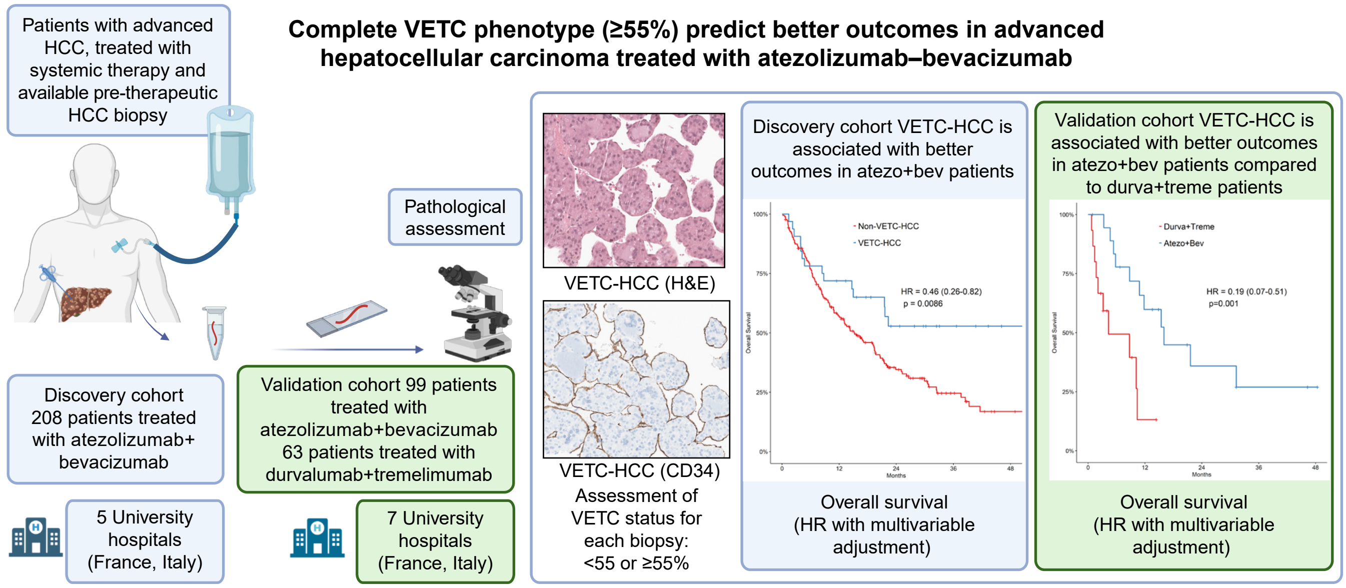

Highlights:

- The complete VETC phenotype predicts response and survival in advanced HCC treated with atezolizumab + bevacizumab.
- In patients with complete VETC-HCC, atezolizumab + bevacizumab led to longer survival than durvalumab + tremelimumab.
- The VETC phenotype is easily assessable in routine practice on biopsy.
- A complete VETC phenotype on pre-treatment biopsy could help guide first-line therapy, favoring atezolizumab–bevacizumab.

Impact and implications:

Routinely assessable histological variables associated with response and survival are still lacking in patients with advanced HCC treated with atezolizumab + bevacizumab. In this study, we identified and validated, in a multicentric series of 371 pre-therapeutic biopsies, the complete VETC phenotype as a predictive factor for PFS and OS in patients with HCC treated with atezolizumab + bevacizumab compared with those treated with durvalumab + tremelimumab. The VETC phenotype is easily assessable in routine practice on biopsy and could help select first-line therapy, although prospective validation of these results remains necessary.

https://doi.org/10.1016/j.jhepr.2026.101874
© 2026 The Author(s). Published by Elsevier B.V. on behalf of European Association for the Study of the Liver (EASL). This is an open access article under the CC BY license (http://creativecommons.org/licenses/by/4.0/). JHEP Reports, 2026, 8, 1–13

JHEP|Reports

Vessels encapsulating tumor clusters predict better outcomes in advanced hepatocellular carcinoma treated with atezolizumab–bevacizumab

Alexandre Sayadi[1,2], Claudia Campani[3,4], Astrid Laurent-Bellue[5], Marianne Ziol[4,6], Luca Di Tommaso[7,8], Etienne Becht[1], Benoit Terris[9], Julien Calderaro[10], Luca Messerini[11,12], Miguel Albuquerque[1,2], Federico Lipsich[13], Carolina Gutierrez[13], Marion Dhooge[14], Lisa Lellouche[14], Giuliana Amaddeo[15], Alina Pascale[16], Olivier Rosmorduc[16], Fabio Marra[3], Lorenza Rimassa[8,17], Marco Dioguardi Burgio[1,13], Jean-Charles Nault[4,18], Mohamed Bouattour[19], Valérie Paradis[1,2], Aurélie Beaufrère[1,2,*]

JHEP Reports **2026**. vol. 8 | 1–13

Check for updates

Background & Aims: Atezolizumab + bevacizumab and durvalumab + tremelimumab are the standard first-line therapies for advanced hepatocellular carcinoma (HCC). Histological variables routinely associated with outcomes remain poorly described. We aimed to assess the prognostic and predictive value of the vessels encapsulating tumor clusters (VETC) phenotype in this setting.

Methods: We retrospectively analyzed 208 patients in a discovery cohort with advanced HCC treated with atezolizumab + bevacizumab and available pre-treatment biopsies, and 163 patients in a validation cohort, including 99 treated with atezolizumab + bevacizumab and 64 with durvalumab + tremelimumab. In addition to baseline clinico-biological variables, we assessed histological features, including the VETC phenotype, defined as complete (≥55% of tumor area), or incomplete (1–54% of tumor area). Prognostic impacts were evaluated using multivariable Cox models.

Results: In the discovery cohort, the complete VETC phenotype (n = 32) displayed the highest objective response rate in the cohort (66% *vs*. 37% in non- or incomplete VETC-HCC, $p = 0.002$), according to the modified RECIST. The complete VETC phenotype was independently associated with better progression-free survival (PFS) (hazard ratio [HR] 0.42, 95% CI 0.26–0.69, $p < 0.001$) and overall survival (OS) (HR 0.46, 95% CI 0.26–0.82, $p = 0.008$). In the validation cohort, complete VETC-HCC was associated with worse outcomes in patients treated with durvalumab + tremelimumab (PFS: HR 2.44, 95% CI 1.12–5.29, $p = 0.024$; OS: HR 3.90, 95% CI 1.66–9.15, $p = 0.002$). Patients with complete VETC-HCC and treated with atezolizumab + bevacizumab had longer survival than patients with complete VETC-HCC and treated with durvalumab + tremelimumab (PFS: HR 0.43, 95% CI 0.17–1.06, $p = 0.07$; OS: HR 0.19, 95% CI 0.07–0.51, $p = 0.001$).

Conclusions: We identified complete VETC as a potential predictive marker of outcomes in patients with advanced HCC and treated with atezolizumab + bevacizumab or durvalumab + tremelimumab. Prospective validation of these findings is warranted.

© 2026 The Author(s). Published by Elsevier B.V. on behalf of European Association for the Study of the Liver (EASL). This is an open access article under the CC BY license (http://creativecommons.org/licenses/by/4.0/).

Introduction

Hepatocellular carcinoma (HCC) is the third leading cause of cancer-related death and the sixth most common cancer worldwide.[1] Despite advances in diagnosis and therapy, HCC prognosis remains poor with a 5-year survival rate below 15%.[2,3] The IMbrave 150 trial demonstrated that the combination of atezolizumab (targeting programmed death-ligand 1 [PD-L1]) and bevacizumab (targeting VEGFA) significantly improves overall survival (OS) and progression-free survival (PFS) compared with sorafenib, thereby establishing atezolizumab/bevacizumab (atezo + bev) as a standard of care in advanced HCC.[4–7] More recently, the HIMALAYA trial demonstrated that the STRIDE regimen (a single priming dose of tremelimumab (anti–CTLA-4) followed by durvalumab (anti–PD-L1) (durva + treme) also improved OS and PFS over sorafenib, offering an additional first-line treatment option alongside atezo + bev.[8–10] Finally, the CheckMate 9DW trial demonstrated superiority of nivolumab + ipilimumab over lenvatinib and sorafenib.[11] Nonetheless, objective response rate (ORR) is limited to ~20–30% of patients, underscoring the need for reliable predictive biomarkers to guide therapeutic selection among available first-line treatments and optimize efficacy.

Although several plasmatic factors, such as IL-6 levels and the neutrophil-to-lymphocyte ratio, have been associated with response, and composite scores such as CRAFITY (C-reactive protein [CRP] and alpha-fetoprotein [AFP] in ImmunoTherapY) have emerged, none have reached clinical validation (*i.e.* companion marker).[12–14] On the molecular scale, an ‘atezo + bev response signature’ (ABRS) has been generated from HCC

* Corresponding author. Address: Department of Pathology, Beaujon Hospital, 100 boulevard du Général Leclerc, Clichy, 92110, France, Tel.: +33 1 40 87 54 63.
E-mail address: aurelie.beaufrere@aphp.fr (A. Beaufrère).
https://doi.org/10.1016/j.jhepr.2026.101874

transcriptomes, with features linked to antitumor immunity.[15] Zeng *et al.*[16] proposed a deep learning model to predict ABRS on HCC biopsy, which was correlated with PFS but not with OS. Recently, single-cell RNA sequencing from patients with HCC treated with sorafenib or atezo +/- bev identified two biologically distinct responder subgroups: immune-competent and angiogenesis-driven, the latter defined by non-immune environment, higher expression of *VEGFA* and reduced expression of vascular endothelial growth factor (*VEGF*) co-receptor neuropilin-1 (*NRP1*).[17] Still, nearly half of patients remain unclassified for response prediction. Nevertheless, these transcriptomic and AI-based biomarkers are still not used and accessible in routine practice, revealing the need for simpler biomarkers, such as histopathological ones.

The vessels encapsulating tumor clusters (VETC) phenotype has attracted growing interest in recent years.[18–21] VETC is a distinct vascular pattern characterized by sinusoid-like vessels encapsulating tumor clusters. It has been associated with angiogenesis, vascular remodeling, immunosuppressive environment, and tumor dissemination.[22–25] In resectable HCC, the VETC phenotype has been associated with adverse prognosis.[18] Among systemic therapies, bevacizumab targets VEGF-driven antiangiogenesis, providing a biological rationale of a potential benefit from regimens that include VEGF blockade, such as atezo + bev and possibly tyrosine kinase inhibitors in VETC-HCC, as suggested by previous reports.[20,26]

The primary aim of this study was to evaluate the prognostic and predictive value of VETC phenotype in advanced HCC treated with atezo + bev. Secondary exploratory objectives were to evaluate the associations between VETC and histo-molecular subtypes, β-catenin/glutamine synthetase (GS) expression, and immune infiltrates on biopsy, and to examine their relationships with clinical outcomes.

Patients and methods

Study population

We conducted a retrospective, multicenter international study including patients treated for an advanced HCC between January 2020 and December 2024. The study population was divided into a discovery and a validation cohort. The discovery cohort included patients treated with atezo + bev as first- or second-line systemic therapy, with or without locoregional treatment, from five centers in France and Italy (Beaujon, Cochin, Henri Mondor, Paul Brousse, and Careggi Hospitals). The validation cohort included patients treated with atezo + bev from two external centers (Avicenne and Humanitas Hospitals), as well as patients treated with durva + treme from five centers (Beaujon, Paul Brousse, Avicenne, Careggi, and Humanitas Hospitals), with or without locoregional therapy.

Inclusion criteria were: patients with histologically confirmed advanced HCC and available HCC biopsy performed before systemic treatment initiation. Exclusion criteria were: age <18 years; absence of tumor biopsy or biopsy performed outside participating centers; monotherapy with atezolizumab, bevacizumab, durvalumab, or tremelimumab; any other systemic therapy in the delay between HCC biopsy and the start of atezo + bev or durva + treme (*i.e.* patients with other systemic conditions before HCC biopsy were included); and fibrolamellar HCC or combined HCC-cholangiocarcinoma.

Written consent was obtained from all patients as required by French and Italian legislation. The study was conducted in accordance with the ethical guidelines of the Declaration of Helsinki, and the study protocol was approved by local ethics committees (CER Paris Nord, No. 2023-227; CEAVC 23673).

Clinical data collection

Clinical, radiological, and biological data were retrospectively extracted from electronic medical records (Supplementary methods).

Endpoints were defined as follows: OS measured from the start of atezo + bev or durva + treme to death from any cause; PFS from the start of atezo + bev or durva + treme to initial diagnosis of progression or death, regardless of cause. For each patient, radiological response was assessed according to modified RECIST (mRECIST)[27] by local hepatobiliary radiologists in routine practice, mainly during multidisciplinary tumor board meetings; no central retrospective rereading of imaging was performed for this study. Responders were defined as patients achieving a complete or partial response (at any time during the follow-up). Non-responders were defined by stable disease or progressive disease. Patients who did not have any radiological evaluation (*i.e.* early death, clinical progression) were considered as non-responders.

Histopathological assessment

All hematoxylin–eosin–saffron (HES)-stained slides were reviewed by two liver pathologists (AB, AS), blinded to clinical data. The following criteria were assessed: tumor grade (according to WHO classification[28]), histological subtype (according to WHO classification,[28] including macrotrabecular-massive [MTM], steatohepatitic [SH], scirrhous [SQ] and clear cell [CC] subtypes; or HCC not otherwise specified [NOS]), VETC phenotype, tumor fibrosis and necrosis, inflammatory infiltrate, tumor steatosis, and tumor cholestasis (Table S1).

Immunohistochemistry

For all patients, when formalin-fixed, paraffin-embedded (FFPE) tissue sections had enough material left, we performed immunostaining with CD34 (ABCAM, QBEND10, AB8536, 1:500).

The VETC phenotype was quantitatively assessed as a percentage of tumor area on HES-stained slide and CD34 immunostained. When CD34 staining was not feasible due to lack of remaining tumor tissue, the VETC phenotype assessed based solely on HES staining, according with previous studies.[29] In case of discordance between HES and CD34 immunostaining, VETC phenotype was assessed on CD34 was retained. The VETC phenotype was finally classified as absent, incomplete (1–54% of tumoral surface with endothelial cells encapsulating tumor clusters) or complete (≥55% of tumoral surface with endothelial cells encapsulating tumor clusters), based on previous work by Renne *et al.*[18]

For Beaujon Hospital's cases in the discovery cohort, supplementary formalin-fixed paraffin-embedded tissue sections were stained with several antibodies, depending on remaining material (Supplementary methods).

Statistical analysis

Continuous variables were expressed as median (1st–3rd quartile) and were compared using the Wilcoxon rank sum test. Categorical variables were expressed as n (%) and compared using Pearson's X^2 test. All tests were two-tailed; p <0.05 was considered statistically significant. No adjustments for multiple comparisons were made. Comparisons involving histological subtypes and secondary biomarkers were considered exploratory analyses.

A weighted kappa coefficient (κ), applying a linear weighting scheme, was computed to assess concordance between VETC phenotype assessment on HES-stained sections and CD34 immunohistochemistry.[30,31] To assess the robustness of the predefined 55% cut-off derived from resection-based VETC quantification, we performed a sensitivity analysis scanning all possible thresholds.[18] For each 5% increment (5–95%), we binarized VETC as ≥threshold% *vs.* <threshold% and fitted separate Cox models for OS. Hazard ratios (HRs), 95% CIs, p values, and the distribution of patients across cut-offs were recorded.

Survival analyses (OS and PFS) were performed using the Kaplan–Meier method. HRs and 95% CIs were estimated using Cox proportional hazards models; p values for individual covariates were obtained from Wald tests. The proportional hazards assumption was assessed by testing for non-significant correlations between event times and Schoenfeld residuals. For multivariable adjusted Cox models, variables with a p value <0.2 in univariable analysis and available data for >80% of patients were retained. In addition, clinically relevant covariates known to influence outcomes—defined *a priori*—were included in all models, irrespective of their statistical significance. These included sex, age (<65/>65 years old), Eastern Cooperative Oncology Group (ECOG) Performance Status, Child–Pugh class, prior locoregional treatment, etiology of liver disease (viral/non-viral), presence of macrovascular invasion or extrahepatic spread, and serum AFP >400 ng/ml. In addition, to explore whether VETC acted as a predictive rather than purely prognostic biomarker, we fitted Cox proportional hazards models including treatment group, VETC status, and their interaction term (treatment × VETC), as proposed by Ballman for distinguishing predictive from prognostic biomarkers.[32] A statistically significant interaction term (p_interaction <0.05) was interpreted as evidence that the association between VETC and outcome differed according to treatment regimen.

All statistical analyses were performed using R software (version 4.3.3, R Foundation for Statistical Analysis, Vienna, Austria). Survival analyses were conducted with the survival and survminer packages, interaction analyses with survival and emmeans, and descriptive and regression tables were generated using gtsummary. Figures were produced with ggplot2.

Results

Patient characteristics

Three hundred and seventy-one patients met inclusion criteria and were included for further analysis divided into 208 patients in the discovery cohort and 163 patients in the validation cohort (99 patients treated with atezo + bev and 64 patients with durva + treme) (Fig. S1). Baseline characteristics for discovery and validation cohorts are summarized in Table 1. The most frequent etiology was viral hepatitis (*i.e.* HBV/HCV, 45%, n = 94 and 51%, n = 83, respectively), followed by metabolic dysfunction-associated steatotic liver disease (MASLD) (23%, n = 48 and 20%, n = 33, respectively). The majority of patients had extrahepatic spread and/or macroscopic vascular invasion (*i.e.* Barcelona Clinic Liver Cancer [BCLC] C, 65%, n = 136 and 66%, n = 108) CRP (2.2 *vs.* 11 mg/dl, p <0.001) and CRAFITY score (31% with high score *vs.* 15%, p = 0.025) were significantly higher in the validation cohort, but CRP and therefore CRAFITY score were available in only 79 patients (48%) in the validation cohort. Regarding previous therapy, 76% (n = 159) and 74% (n = 122) of patients, respectively, did not receive locoregional therapy before atezo + bev or durva + treme treatment. Median duration of follow-up was 29.7 months (range, 0.8–56.1 months) in the discovery cohort and 26.7 months (range, 1.0–60.3 months) in the validation cohort. Median PFS was 6.8 months (95% CI 6.1–9.2) and 6.6 months (95% CI 5.1–8.3), respectively. Median OS was 17.1 months (95% CI 13.6–21.5), and 14.8 months (95% CI 12.2–17.7), respectively. In discovery cohort, median delay between biopsy and atezo + bev initiation was 1.16 months (IQR 0.7–5.1 months). In validation cohort, median delay between biopsy and systemic treatment initiation was 3.0 months (IQR 0.6–10.2 months).

Correlation between clinico-biological features, response, and survival in the discovery cohort

In univariate analysis, median PFS and OS were significantly shorter in patients having a baseline serum AFP >400 ng/ml than patients with serum AFP <400 ng/ml (PFS: 4.7 *vs.* 9.7 months, HR: 1.58, 95% CI 1.17–2.15, p = 0.003; OS: 12.4 *vs.* 19.7 months, HR: 1.49, 95% CI 1.05–2.11, p = 0.026) (Fig. S2). CRAFITY score was available for 166 patients. CRAFITY score significantly stratified patients on PFS (high-risk patients *vs.* low-risk patients, median PFS 3.3 months *vs.* 11.7 months, univariable HR: 3.07, 95% CI 1.99–4.73, p <0.001) and OS (high-risk patients *vs.* low-risk patients, median OS 8 months *vs.* 32.4 months, univariable HR: 3.44, 95% CI 2.05–5.77, p <0.001) (Fig. S2). Moreover, 41% of patients (n = 86) were classified as responders according to mRECIST. Among responders, median OS was 41.6 months compared with 9.7 months in non-responders (univariable HR 0.21, 95% CI 0.14–0.32, p <0.001) (Fig. S3).

Impact of histological subtypes on response and survival in the discovery cohort

The main histological characteristics and correlation with response are summarized in Table S2. Regarding quality criteria for HCC biopsy, median length of HCC tissue on biopsy was 11 mm (IQR 6–15 mm). One hundred and one (49%) HCC were classified as NOS-HCC, 58 (28%) MTM-HCC, 24 (12%) steatohepatitic (SH)-HCC, 16 (8%) scirrhous (SQ)-HCC and nine (4%) clear cell (CC)-HCC. NOS-HCC was associated with higher ORR (56% [n = 48] NOS-HCC among responders *vs.* 41% [n = 50] among non-responders, p = 0.035). When compared with NOS-HCC, MTM-HCC was associated with lower ORR (32.8% [n = 19/58] *vs.* 49.5% [n = 50/101], p = 0.04, Fig. 1). No significant association with ORR was observed for the other histological subtypes.

Table 1. Baseline patients' characteristics.

Characteristic	Discovery cohort, n = 208*	Validation cohort, n = 163*	*p* value†
Age (years)	69.5 (61.0–77.0)	68.0 (61.0–76.0)	0.4
Sex			0.12
Male	183 (88)	134 (82)	
Female	25 (12)	29 (18)	
Alcohol-related liver disease (ALD)	31 (15)	29 (18)	0.5
Metabolic dysfunction-associated steatotic liver disease (MASLD)	48 (23)	33 (20)	0.5
Metabolic dysfunction and alcohol-related liver disease (MetALD)	33 (16)	19 (12)	0.2
Viral hepatitis (HBV/HCV)	94 (45)	83 (51)	0.3
ECOG PS‡			0.056
0	123 (59)	76 (49)	
≥1	85 (41)	79 (51)	
Macroscopic vascular invasion	87 (42)	64 (39)	0.6
Extrahepatic spread	74 (36)	59 (36)	0.9
BCLC stage			0.9
B	72 (35)	55 (34)	
C	136 (65)	108 (66)	
Child–Pugh class			0.3
No cirrhosis	76 (37)	62 (38)	
A	105 (50)	88 (54)	
B	27 (13)	13 (8.0)	
AFP (ng/ml)§	71.0 (6.0–3,858.0)	124.5 (9.4–3,437.0)	0.4
AFP >400 ng/ml§	81 (40)	66 (41)	0.8
CRP (mg/L)¶	11.0 (5.0–31.0)	2.2 (0.7–8.7)	<0.001
CRAFITY score**			0.025
Low	48 (29)	30 (38)	
Intermediate	66 (40)	37 (47)	
High	52 (31)	12 (15)	
Previous locoregional therapy (TACE and/or TARE within 6 months)‡	49 (24)	41 (26)	0.8
Systemic treatment received			
Durvalumab + tremelimumab	0 (0)	64 (39)	
Atezolizumab + bevacizumab	208 (100)	99 (61)	

AFP, alpha-fetoprotein; BCLC, Barcelona Clinic Liver Cancer; CRAFITY, CRP and AFP in ImmunoTherapY; ECOG, Eastern Cooperative Oncology Group; TACE, transarterial chemoembolization; TARE, transarterial radioembolization.
*Data are presented as median (Q1–Q3) or n (%).
†Wilcoxon rank sum test; Pearson's Chi-squared test.
‡Available for 363 cases.
§Available for 367 cases.
¶Available for 248 cases.
**Available for 245 cases.

In multivariable analysis, median PFS and OS in patients with MTM-HCC were 4.7 months and 13.2 months, respectively, but were not statistically different from patients with NOS-HCC (9 months, HR: 1.37, 95% CI 0.94–2.00, p = 0.1 and 21.5 months, HR: 1.32, 95% CI 0.85–2.06, p = 0.2, respectively) (Fig. 1 and 2). Median PFS and OS in SQ-HCC were 6.4 months and 10.8 months, respectively (HR 1.57, 95% CI 0.90–2.76, p = 0.1 and HR 1.60, 95% CI 0.84–3.05, p = 0.149, respectively) (Fig. 2; Fig. S4). CC-HCC was associated with shorter PFS than NOS-HCC (median PFS 6 months, HR: 2.26, 95% CI 1.07–4.77, p = 0.032) (Fig. 2; Fig. S4). SH-HCC did not impact PFS nor OS in comparison to NOS-HCC (Fig. 2; Fig. S4).

The complete VETC phenotype is strongly associated with response and survival in the discovery cohort

Assessment of VETC phenotype on CD34 stain was blinded of VETC phenotype assessed on HES stain. In 162 HCC biopsies (78%), CD34 stain was available. Agreement between CD34 and HES stain for VETC phenotype was strong (weighted kappa = 0.89, Tables S3 and S4).

The complete VETC phenotype (*i.e.* VETC-HCC according to Renne *et al.*[18]) was present in 32 HCC biopsies (15%) (23 diagnosed on HES and CD34 immunohistochemistry and nine on HES alone [Fig. S5]); while 25 patients (12%) had incomplete VETC phenotype (Fig. 3). In complete VETC-HCC, the mean percentage of tumor area with tumoral clusters circumscribed by CD34+ endothelial cells was 75%. Main clinical, biological, and histological characteristics of patients with VETC-HCC are shown in Table 2. VETC-HCC were mainly MTM-HCC (44%) or NOS-HCC (50%), without any SH-HCC or SQ-HCC. Besides histological subtypes, there was not any clinical, biological, nor other histological difference between non- or incomplete VETC and VETC-HCC. The best ORR was seen among patients with VETC-HCC, with 21 (66%) responders compared with 65 (37%) responders in patients with non- or incomplete VETC-HCC (p = 0.002). In univariable analysis, median PFS in patients with VETC-HCC was 13.7 months compared to 6.7 months in patients with non- or incomplete VETC-HCC (HR: 0.53, 95% CI 0.34–0.83, p = 0.005). Median OS in patients with VETC-HCC was 51.2 months compared with 15.2 months in patients with non- or

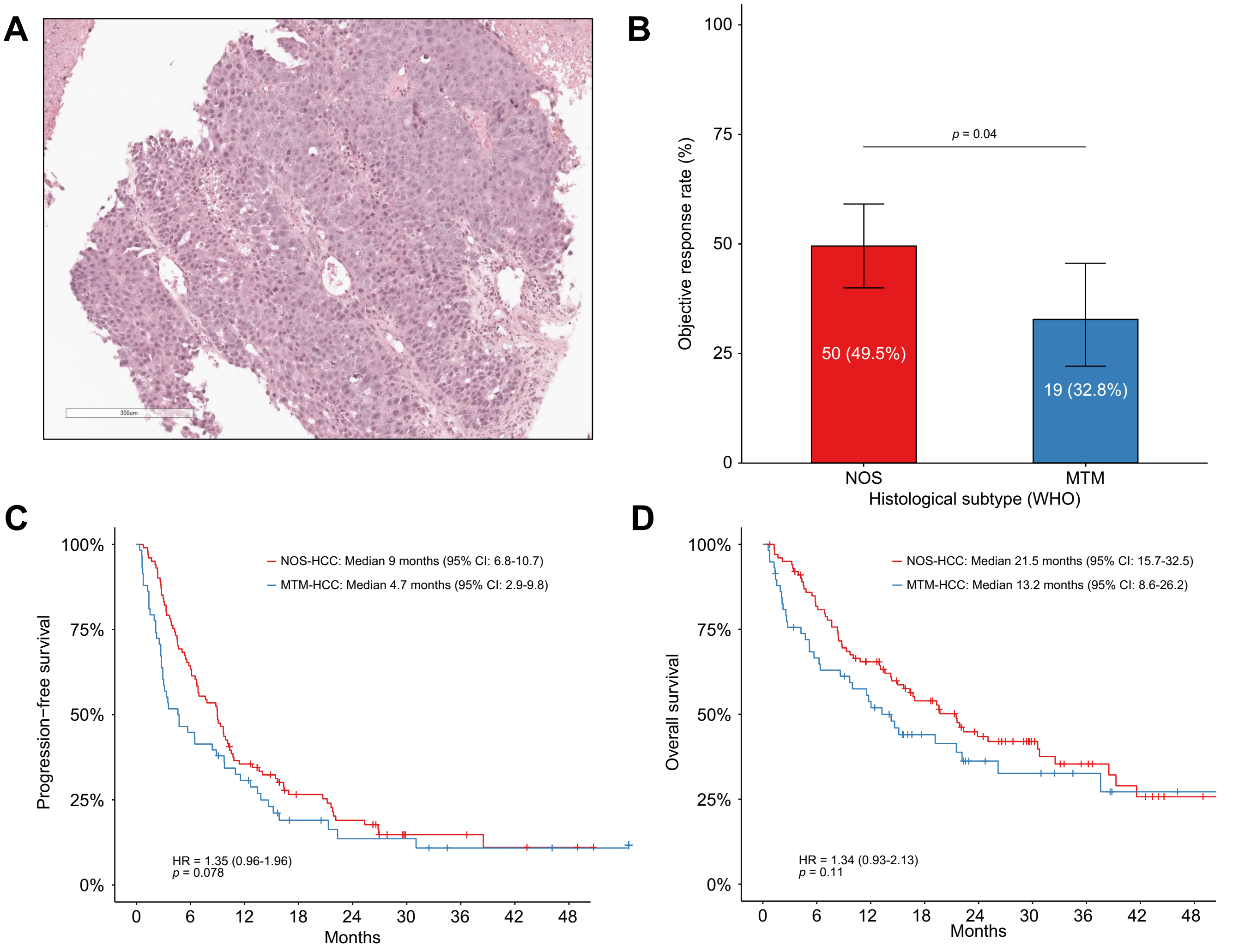

N° at risk									
NOS-HCC	101	64	35	21	15	5	5	3	2
MTM-HCC	58	26	17	8	5	5	2	2	1

N° at risk									
NOS-HCC	101	80	61	45	31	20	13	8	4
MTM-HCC	58	37	29	17	11	9	6	3	2

Fig. 1. Prognostic impact of MTM-HCC in the discovery cohort. (A) MTM-HCC, HES stain, × 100. (B) Objective response rate (ORR) according to mRECIST in NOS-HCC and MTM-HCC. Pearson's X^2 test. (C) Progression-free survival in patients with NOS-HCC and MTM-HCC. (D) Overall survival in patients with NOS-HCC and MTM-HCC. HRs from univariate Cox regression are displayed with their 95% CI and *p* value (Wald test). HES, hematoxylin–eosin–saffron; HR, hazard ratio; mRECIST, modified RECIST; MTM-HCC, macrotrabecular-massive hepatocellular carcinoma; NOS-HCC, not otherwise specified hepatocellular carcinoma.

incomplete VETC-HCC (HR: 0.53, 95% CI 0.31–0.91, p = 0.022) (Fig. 4).

Patients with incomplete VETC-HCC had similar prognosis than those with non–VETC-HCC: median PFS, 7.8 months *vs*. 6.7 months (HR 1.11, 95% CI 0.71, –1.76, p = 0.63); median OS, 13.1 months *vs*. 16.4 months (HR 1.07, 95% CI 0.63–1.82, p = 0.79). When considering only the 162 HCC with available CD34 stain, the complete VETC phenotype still significantly stratified patients on PFS (VETC-HCC, median 15.8 months; incomplete VETC-HCC, 6.4 months; non–VETC-HCC 6.5 months; HR VETC-HCC *vs*. incomplete VETC-HCC: 0.38, 95% CI 0.19–0.77, p = 0.007) (Fig. S6). Clinical, biological and histological characteristics of HCC with VETC images (incomplete and complete VETC-HCC combined) *vs*. without VETC images are summarized in Table S5. Presence of VETC images was associated with GS expression and/or nuclear β-catenin expression (41% *vs*. 17%, p = 0.005) and less T cells infiltrate (median CD8+ T cells/mm^2, 45.6 *vs*. 74.2, p = 0.066; median CD3+ T cells/mm^2, 157.1 *vs*. 285.5, p = 0.04).

Sensitivity analyses evaluating alternative VETC thresholds (5–95% in 5% increments) showed that the prognostic signal (PFS) was maximal and most stable between 50% and 60%, with the 55% cut-off yielding the lowest p value (HR 0.53, 95% CI 0.34–0.83, p = 0.005) (Table S6, Fig. S7).

Impact of *CTNNB1*, *TP53* status, and inflammatory infiltrate in the discovery cohort

CD8+ infiltrate was assessable in 106 (51%) patients. Median density was 62 CD8+ T cells/mm^2. When splitting on median density, high CD8+ infiltrate was not significantly associated with longer PFS (HR 0.70, 95% CI 0.46–1.05, p = 0.086), nor OS

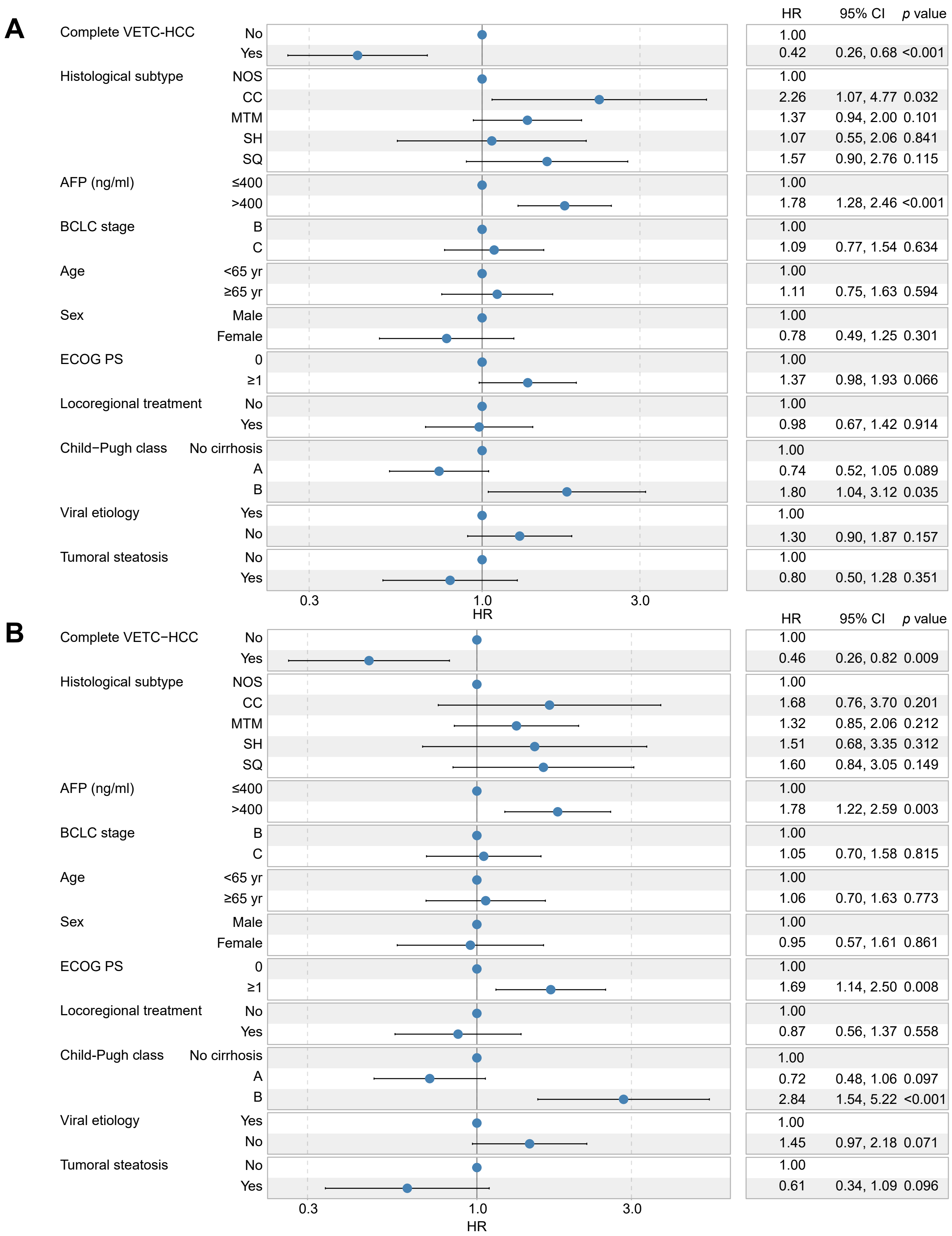

Fig. 2. Multivariable Cox regression for progression-free survival and overall survival in the discovery cohort. AFP, alpha-fetoprotein; BCLC, Barcelona Clinic Liver Cancer; ECOG PS, Eastern Cooperative Oncology Group Performance status; NOS, not otherwise specified; CC, clear cell; HR, Hazard ratio; MTM, macro-trabecular-massive; SH, steatohepatitic; SQ, scirrhous; HCC, hepatocellular carcinoma; VETC, Vessels Encapsulating Tumor Clusters.

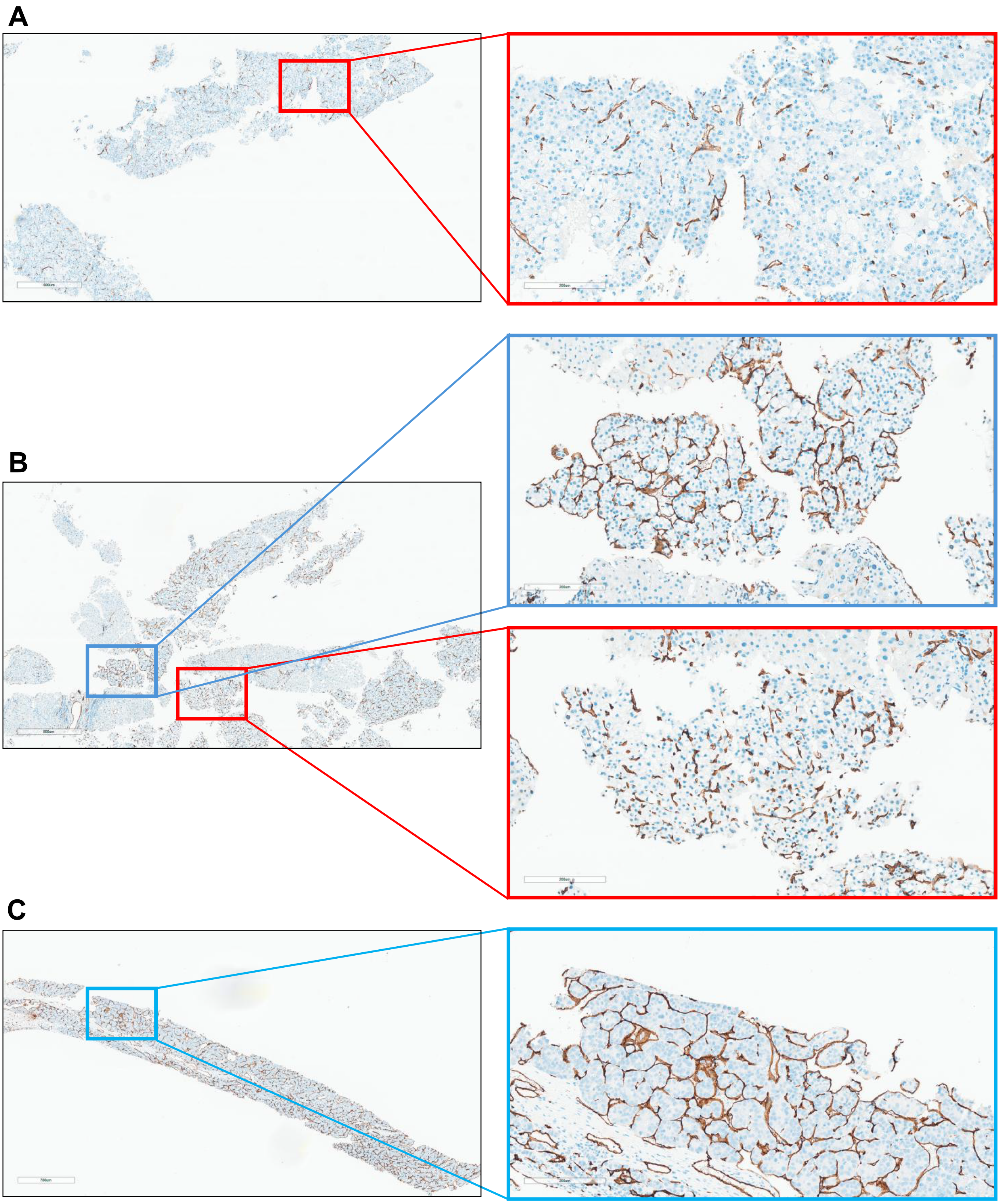

Fig. 3. Histological view of incomplete and complete VETC phenotype in HCC biopsies. (A) Non–VETC-HCC, CD34 immunostaining (left, × 40; right, × 100). Higher magnification (red box) shows only capillary pattern, without any VETC images. (B) Incomplete VETC-HCC, CD34 immunostaining (left, × 30; right, × 150). This HCC biopsy associates minor areas (<55%) of VETC images (high magnification, blue box); with majority of non-VETC images (high magnification, red box). Pearson's X^2 test. (C) Complete VETC-HCC, CD34 immunostaining (left, × 30; right, × 150). Formation of VETC was seen on majority of tumor area (≥55%, red box). HCC, hepatocellular carcinoma; VETC, vessels encapsulating tumor clusters.

Table 2. Characteristics of VETC-HCC, compared with incomplete and No–VETC-HCC in the discovery cohort.

Characteristic	VETC-HCC, n = 32*	Incomplete/non–VETC-HCC, n = 176*	*p* value†
Age (years)	70.0 (61.0–77.0)	68.5 (62.0–76.5)	0.9
Sex (%)			0.8
Male	29 (91)	154 (88)	
Female	3 (9.4)	22 (13)	
Viral hepatitis (HBV/HCV) (%)	13 (41)	80 (45)	0.6
Child–Pugh class (%)			0.4
No cirrhosis	15 (47)	61 (35)	
A	13 (41)	92 (52)	
B	4 (13)	23 (13)	
ECOG PS (%)			0.5
0	17 (53)	106 (60)	
≥ 1	15 (47)	70 (40)	
Macrovascular invasion (%)	18 (56)	69 (39)	0.072
Extrahepatic spread (%)	10 (31)	64 (36)	0.6
BCLC stage (%)			0.7
B	10 (31)	62 (35)	
C	22 (69)	114 (65)	
AFP‡	111.5 (6.0–3,000.0)	54.0 (6.0–5,077.0)	0.8
CRP§	12.0 (5.0–36.0)	8.0 (6.0–23.0)	0.7
CRAFITY (%)¶			0.6
Low	9 (35)	39 (28)	
Intermediate	8 (31)	58 (41)	
High	9 (35)	43 (31)	
Macrotrabecular-massive-HCC (%)	14 (44)	44 (25)	0.030
Steatohepatitic-HCC (%)	0 (0)	24 (14)	0.030
Scirrhous-HCC (%)	0 (0)	16 (9.1)	0.14
Clear cell-HCC (%)	2 (6.3)	7 (4.0)	0.6
Not otherwise specified-HCC (%)	16 (50)	82 (47)	0.7
p53 expression** (%)	4 (27)	30 (33)	0.8
PD-L1 >0 (TPS and/or CPS) (%)††	4 (27)	29 (41)	0.3
B-catenin expression (%)‡‡	5 (29)	23 (23)	0.6
CD8+ T cells (/mm^2)**	50.3 (31.0–85.8)	70.8 (28.1–188.0)	0.5
CD3+ T cells (/mm^2)§§	191.6 (114.1–313.4)	251.4 (104.7–494.4)	0.5
ORR (%)			0.002
No response	11 (34)	111 (63)	
Response	21 (66)	65 (37)	

AFP, alpha-fetoprotein; BCLC, Barcelona Clinic Liver Cancer; CPS, combined positive score; CRAFITY, CRP and AFP in ImmunoTherapY; CRP, C-reactive protein; ECOG, Eastern Cooperative Oncology Group; HCC, hepatocellular carcinoma; PD-L1, programmed death-ligand 1; TPS, tumor proportion score; VETC, vessels encapsulating tumor clusters.
*Data are presented as median (Q1–Q3) or n (%).
†Wilcoxon rank sum test; Pearson's Chi-squared test.
‡Available for 205 cases.
§Available for 169 cases.
¶Available for 166 cases.
**Available for 106 cases.
††Available for 86 cases.
‡‡Available for 117 cases.
§§Available for 100 cases.

(HR 0.81, 95% CI 0.51–1.28, *p* = 0.38) (Fig. S8). CD3 infiltrate was assessable in 100 (48%) patients. Median density was 240 CD3+ T cells/mm^2. Splitting on median density did not reveal any prognostic impact (PFS, HR 0.74, 95% CI 0.49–1.13, *p* = 0.17; OS, HR 0.83, 95% CI 0.52–1.33, *p* = 0.44).

TP53 and *CTNNB1* status (through p53 and GS/β-catenin expression) was assessable in 103 (50%) patients. Among these 103 patients, 32 (31%) had p53 overexpression or loss of expression (*i.e.* p53-mutated profile), whereas 25 (24%) showed diffuse GS expression and/or nuclear β-catenin expression and three (3%) had both p53-mutated profile and diffuse GS expression and/or β-catenin expression (Table S7). Neither *TP53* nor *CTNNB1* status was correlated with PFS nor OS (Fig. S8).

Multivariable analysis in the discovery cohort

Results from univariable and multivariable analysis of factors associated with PFS and OS are displayed in Table S8 and Fig. 2. AFP >400 ng/ml, Child–Pugh class B and ECOG Performance Status ≥ 1 were associated with shorter PFS and OS. The complete VETC phenotype remains associated with longer PFS and OS in multivariable analysis: HR 0.42 (95% CI 0.26–0.68, *p* <0.001) and 0.46 (95% CI 0.26–0.82, *p* = 0.009), respectively. CC-HCC was associated with shorter PFS (HR 2.26, 95% CI 1.07–4.77, *p* = 0.032). Non-viral etiology was not significantly associated with shorter PFS (HR 1.39, 95% CI 0.98–1.97, *p* = 0.065) and was associated with shorter OS (HR 1.65, 95% CI 1.11–2.45, *p* = 0.014).

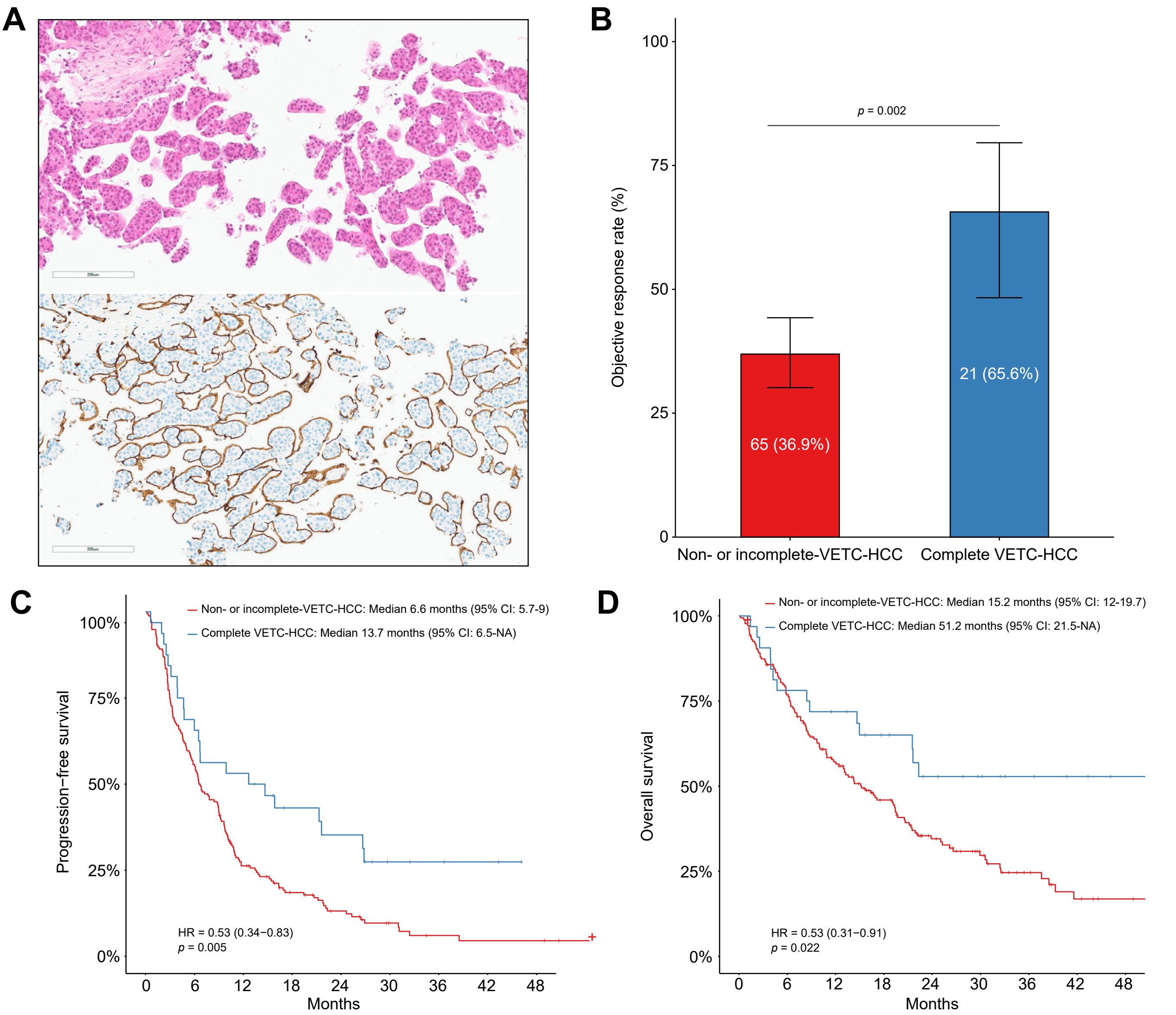

N° at risk									
Non- or incomplete -VETC-HCC	176	98	44	26	16	8	4	3	3
Complete VETC-HCC	32	21	17	11	9	4	3	2	0

N° at risk									
Non- or incomplete -VETC-HCC	176	131	92	64	39	25	15	8	5
Complete VETC-HCC	32	25	22	17	12	9	6	4	2

Fig. 4. Prognostic impact of complete VETC phenotype (≥55%) in the discovery cohort. (A) HCC with complete VETC phenotype (*i.e.* complete VETC-HCC), HES, and CD34 stains, × 200. Tumoral clusters are circumscribed by endothelial cells expressing CD34. (B) Objective response rate (ORR) according to mRECIST in patients with non- or incomplete VETC-HCC (red) and complete VETC-HCC (blue). (C) Progression-free survival in patients with non- or incomplete VETC-HCC and complete VETC-HCC. (D) Overall survival in patients with non- or incomplete VETC-HCC and complete VETC-HCC. HRs from univariate Cox regression are displayed with their 95% CI and *p* value (Wald test). HCC, hepatocellular carcinoma; HES, hematoxylin–eosin–saffron; HR, hazard ratio; mRECIST, modified RECIST; VETC, vessels encapsulating tumor clusters; mRECIST, modified Response Evaluation Criteria in Solid Tumors.

Assessment of predictive effect of VETC-HCC phenotype in patients treated with atezo + bev

In univariable analysis, complete VETC-HCC was associated with shorter OS (HR = 4.22, 95% CI 1.53–11.64, *p* = 0.005) in patients treated with durva + treme compared with those treated with atezo + bev, without difference on PFS (HR = 1.35, 95% CI 0.60–3.05, *p* = 0.47). No treatment-related differences were observed in patients with non- or incomplete VETC-HCC (PFS: HR 0.72, 95% CI 0.47–1.11, *p* = 0.13 and OS: HR 0.81, 95% CI 0.49–1.34, *p* = 0.4) (Fig. 5).

In multivariable analysis, among patients with non- or incomplete VETC-HCC, no difference in PFS or OS was observed between patients treated with atezo + bev and those treated with durva + treme (PFS: HR 1.52, 95% CI 0.93–2.48, *p* = 0.095 and OS: HR 1.22, 95% CI 0.69–2.13, *p* = 0.5). Complete VETC-HCC had a negative prognostic role in durva + treme patients (n = 64) (PFS: HR 2.44, 95% CI

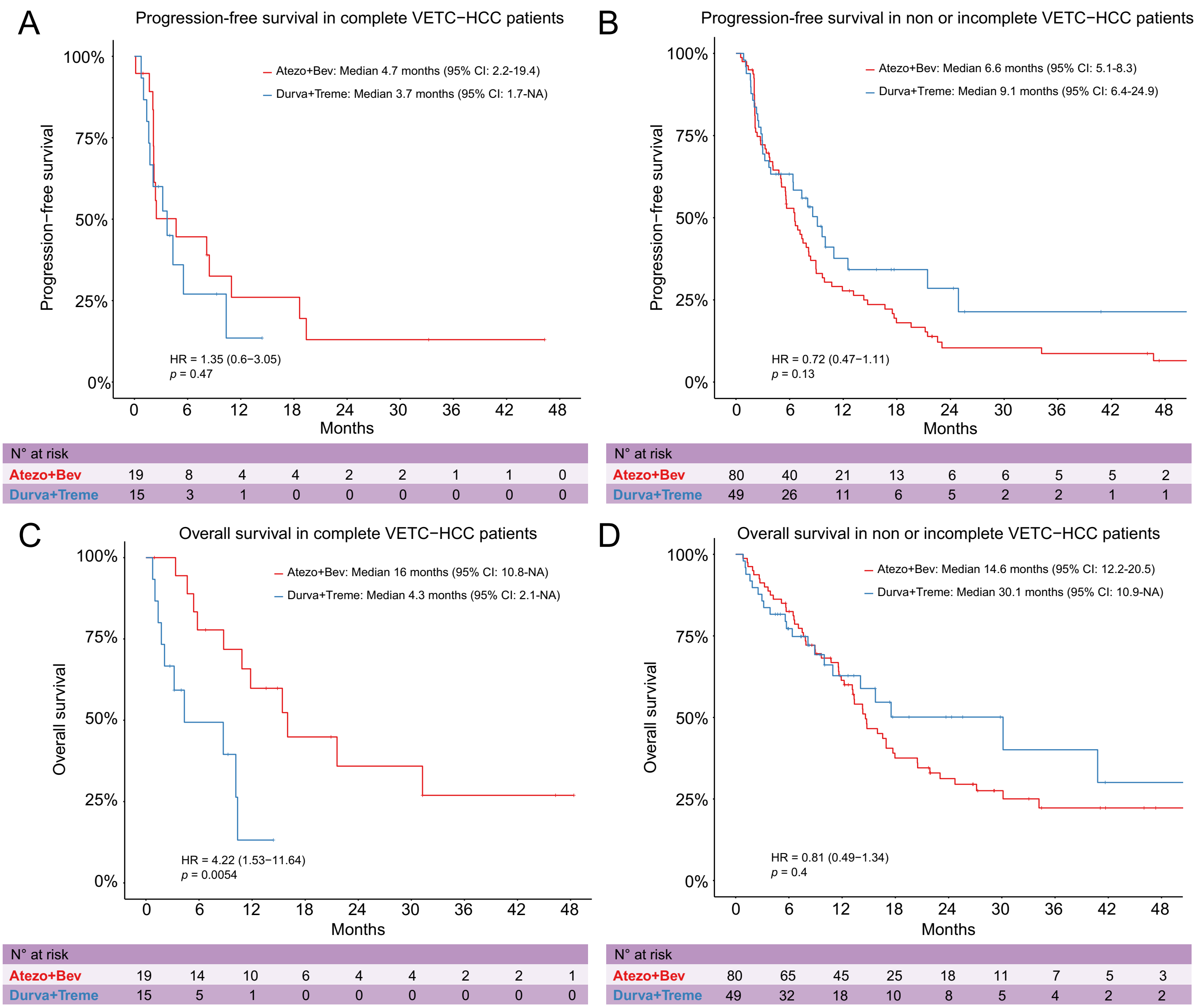

Fig. 5. Predictive impact of VETC phenotype on survival of patients treated with atezo + bev or durva + treme. (A) PFS according to atezo + bev (red) or durva + treme (blue) treatments in patients with complete VETC-HCC. (B) PFS according to atezo + bev (red) or durva + treme (blue) treatments in patients with non or incomplete VETC-HCC. (C) OS according to atezo + bev (red) or durva + treme (blue) treatments in patients with complete VETC-HCC. D: OS according to atezo + bev (red) or durva + treme (blue) treatments in patients with non- or incomplete VETC-HCC. HRs from univariate Cox regression are displayed with their 95% CI and *p* value (Wald test). atezo + bev, atezolizumab–bevacizumab; durva + treme, durvalumab-tremelimumab; HCC, hepatocellular carcinoma; HR, hazard ratio; PFS, progression-free survival; VETC, vessels encapsulating tumor clusters; OS, overall survival.

1.12–5.29, p = 0.024 and OS: HR 3.90, 95% CI 1.66–9.15, p = 0.002). Patients with complete VETC-HCC and treated with atezo + bev had longer survival than patients with complete VETC-HCC and treated with durva + treme (PFS: HR 0.43, 95% CI 0.17–1.06, p = 0.07; OS: HR 0.19, 95% CI 0.07–0.51, p = 0.001). Among patients treated with durva + treme, complete VETC-HCC was associated with poorer outcomes (PFS: HR 2.44, 95% CI 1.12–5.29, p = 0.024; OS: HR 3.90, 95% CI 1.66–9.15, p = 0.002). The interaction term VETC-HCC × treatment was significant, highlighting the predictive role of VETC-HCC (PFS: $HR_{\_interaction}$ = 0.28, 95% CI 0.10– 0.79, p = 0.016; OS: $HR_{\_interaction}$ = 0.15, 95% CI 0.05–0.48, p = 0.001) (Table S9). These results were confirmed in the whole cohort (Fig. S9, Table S10). Sensitivity analyses restricted to patients with OS ≥3 months showed comparable results (Table S11).

Discussion

Despite growing interest in predictive biomarkers for systemic therapy in HCC, robust predictors of treatment response are still lacking. In this study, we first identified the complete VETC phenotype as a prognostic factor independently associated with improved PFS and OS in patients treated with atezo + bev. In the validation cohort, we demonstrated a predictive effect of complete VETC phenotype, with different outcomes

between patients treated with atezo + bev and durva + treme. In patients treated with atezo + bev, complete VETC-HCC was associated with longer OS without difference in PFS. This discrepancy could be partially attributable to more noise and early events in a smaller cohort than discovery cohort. In the whole cohort (discovery and validation), benefit from atezo + bev in complete VETC-HCC was significant, with longer PFS and OS. In contrast, in patients treated with durva + treme, complete VETC-HCC was associated with shorter OS; given the limited size of this subgroup, this estimate should be interpreted cautiously and considered exploratory. Overall, these findings are in line with previous works reporting more benefit from tyrosine kinase inhibitors (TKI)-based and bevacizumab-based regimens compared with dual immunotherapy in patients with unresectable VETC-HCC.[26]

The molecular underpinnings of VETC-HCC have been increasingly elucidated. Zhou *et al.*[25] demonstrated the pivotal role of angiopoietin-2 (Ang2) in VETC formation, regulated in part by androgen receptor signaling. VETC-HCCs display elevated expression of fibroblast growth factor (FGF) and its receptor FGF-R3/4, while VEGFA and VEGF-R2 expression levels are comparable to non–VETC-HCC.[21,33,34] Moreover, the VETC phenotype is associated with an immunosuppressive tumor microenvironment, including reduced T-cell infiltration, increased PD-L1 expression, and enrichment of immunosuppressive macrophages, as TREM2+ macrophages.[18,23,24,35] These features raise important considerations regarding therapeutic responsiveness in VETC-HCC.[21–23] In our study, VETC images were associated with reduced CD3+ T-cell infiltration and a trend towards reduced CD8+ T-cell infiltration, but without difference on PD-L1 expression in immunostaining.

Evidence from prior studies supports differential treatment responses in VETC-HCC. Fang *et al.*[20] reported that patients with recurrent VETC-HCC derived survival benefit from sorafenib, which targets Vascular Endothelial Growth Factor Receptor (VEGFR), Epithelial growth factor receptor (EGFR), Kit, and platelet derived growth factor receptor (PDGFR). The role of bevacizumab in VETC-HCC remains debated, given the non-elevated VEGFA levels;[21] however, VEGFA overexpression has been noted in VETC-HCC with an MTM subtype, potentially enhancing sensitivity to bevacizumab.[34] Additionally, bevacizumab may stimulate antitumor immunity by inhibiting Treg proliferation and myeloid inflammation, thereby enhancing the efficacy of atezolizumab.[15] Collectively, these mechanisms may explain the susceptibility of VETC-HCC to atezo + bev and provide a biological rationale to hypothesize that VETC-HCC may be relatively less sensitive to immune checkpoint inhibitors (ICI)-only strategies; however, causal mechanisms cannot be inferred from our retrospective data and warrant dedicated translational and prospective clinical studies.

Regarding VETC assessment, it was first described with CD34 immunostaining.[18,36] Nevertheless, the pattern is still easily recognizable on H&E or HES stain, with smooth circumscribed tumoral clusters lined by endothelial cells.[21] We illustrated that VETC assessment with HES and CD34 showed strong agreement. Moreover, questions remains about validity of VETC evaluation on biopsy.[21] Renne *et al.*[18] developed VETC threshold at 55% using six tissue microarrays (TMAs) of 1–2 mm diameter per HCC and showed strong correlation between VETC phenotype assessment on TMA and on whole-slide images. Our findings suggest that VETC phenotype is also assessable on HCC biopsy sample and that this cut-off remains useful to stratify patients. These findings suggest that the complete VETC phenotype may help to identify patients who are more likely to respond to anti-VEGF/anti-PD-L1 therapy and therefore optimize treatment efficacy and resource allocation.

Our study also examined histological subtypes and immunohistochemical markers. Notably, MTM-HCC was prevalent in our cohort (28%), higher than reported in surgical series (7–10%).[18,37,38] MTM-HCC is associated with adverse prognostic impact in resectable HCC.[18,37,39] Nevertheless, MTM subtype was not significantly associated with ORR, nor PFS or OS in multivariable analysis.

CRAFITY score significantly stratified patients for PFS and OS in the discovery cohort, consistent with the original study. CRAFITY score was available in <50% of patients in the validation cohort and was therefore not included in multivariable analysis.

This study has several limitations, chiefly its retrospective design, which, even with adjustment for known clinical and biological confounders, makes it difficult to fully account for all potential sources of bias. Even if HCC biopsy is increasingly justified and more widely performed in clinical practice, selection bias may have arisen because of biopsy availability, potentially contributing to the relatively low prevalence of cirrhosis in our cohort.[40] Furthermore, HCC biopsy is inherently subject to sampling variability and limited size in some cases and may not fully reflect tumor heterogeneity. Although prior studies have shown strong concordance between biopsy and resection specimens for transcriptomic analyses, it remains unclear whether this concordance extends to morphological features, including HCC subtypes and the VETC phenotype.[15,41] This question is particularly challenging to resolve in advanced HCC, as the vast majority of patients are not candidates for surgical resection, limiting opportunities for direct comparison. Radiological response was assessed according to mRECIST by local radiologists without central retrospective reread, which may introduce some variability in response classification. However, our main conclusions rely primarily on OS, a hard endpoint that is not influenced by the response assessment method and is particularly relevant in this population with advanced HCC. The durva + treme cohort was relatively small, resulting in a limited number of events relative to the number of covariates in the adjusted Cox models. Consequently, the subgroup-specific HRs estimated in this group are likely to be imprecise and should be interpreted as exploratory. In contrast, the interaction analyses conducted in the pooled validation population provide a more robust assessment of the predictive value of the VETC phenotype. Given the modest sample size and the number of tissue parameters explored, our analyses, particularly for secondary biomarkers, should be regarded as exploratory and hypothesis-generating, and will require confirmation in larger, prospective independent series. Complete VETC-HCC emerges as a candidate predictive biomarker to drive the choice of treatment regimen between atezo + bev and durva + treme, but its clinical utility for treatment selection must be confirmed prospectively

before implementation, through prospective therapeutic trials stratifying first-line treatment based on VETC status.

In conclusion, our multicentric cohort of over 370 pre-treatment biopsies represents one of the largest series to date in patients treated with systemic immunotherapy combinations including atezo + bev and durva + treme. The VETC phenotype, when present in ≥55% of the tumor area, was associated with improved ORR, PFS, and OS in patients treated with atezo + bev compared with those treated with durva + treme. Our findings highlight the potential of VETC phenotype, which is easily assessed in routine clinical practice, as a predictive biomarker in advanced HCC. Further prospective studies are warranted to validate these results moving toward more personalized treatment strategies.

Affiliations

[1]*Université Paris Cité, Centre de recherche sur l'inflammation, INSERM U1149 Paris, France;* [2]*Department of Pathology, AP-HP.Nord, Hôpital Beaujon, FHU MOSAIC*[2]*, SIRIC InsiTu, Clichy, France;* [3]*Department of Experimental and Clinical Medicine, Internal Medicine and Hepatology Unit, University of Florence, Florence, Italy;* [4]*Centre de Recherche des Cordeliers, Sorbonne Université, Inserm, Université de Paris Cité, Team «Functional Genomics of Solid Tumors», Paris, France;* [5]*Department of Pathology, AP-HP Paris-Saclay, Hôpital Bicêtre, Le Kremlin-Bicêtre, France;* [6]*Department of Pathology and biobank BB-0033-00027, APHP.Hôpitaux universitaires Paris Seine-Saint-Denis Hôpital Avicenne, Bobigny, France;* [7]*Pathology Unit, IRCCS Humanitas Research Hospital, Rozzano, Milan, Italy;* [8]*Department of Biomedical Sciences, Humanitas University, Pieve Emanuele, Milan, Italy;* [9]*Department of Pathology, AP-HP Paris Centre, Hôpital Cochin, Paris, France;* [10]*Department of Pathology, Henri Mondor-Albert Chenevier University Hospital, AP-HP, Créteil, France;* [11]*Department of Human Pathology and Oncology, University of Florence, Florence, Italy;* [12]*Department of Oncology, University of Florence, Florence, Italy;* [13]*Department of Radiology, AP-HP Nord, Hôpital Beaujon, Clichy, France;* [14]*Department of Digestive Oncology, AP-HP Paris Centre, Hôpital Cochin, Paris, France;* [15]*Department of Hepatology, Henri Mondor-Albert Chenevier University Hospital, AP-HP, Créteil, France;* [16]*Hepato-Biliary Department, Paul Brousse Hospital, AP-HP Paris-Saclay, Villejuif, France;* [17]*Humanitas Cancer Center, IRCCS Humanitas Research Hospital, Rozzano, Milan, Italy;* [18]*Liver unit, APHP.Hôpitaux universitaires Paris Seine-Saint-Denis Hôpital Avicenne, Bobigny, France;* [19]*Department of Digestive Oncology, AP-HP.Nord, Hôpital Beaujon, Clichy, France*

Abbreviations

ABRS, atezolizumab–bevacizumab response signature; AFP, alpha-fetoprotein; ALD, alcohol-associated liver disease; atezo + bev, atezolizumab–bevacizumab; BCLC, Barcelona Clinic Liver Cancer; CC-HCC, clear cell hepatocellular carcinoma; CPS, combined positive score; CRAFITY, CRP and AFP in ImmunoTherapY; CRP, C-reactive protein; durva + treme, durvalumab-tremelimumab; ECOG, Eastern Cooperative Oncology Group; FGF, fibroblast growth factor; GS, glutamine synthetase; HCC, hepatocellular carcinoma; HES, hematoxylin–eosin–saffron; HR, hazard ratio; MASLD, metabolic dysfunction-associated steatotic liver disease; MetALD, metabolic and alcohol-associated liver disease; mRECIST, modified RECIST; MTM-HCC, macrotrabecular-massive hepatocellular carcinoma; NOS-HCC, not otherwise specified hepatocellular carcinoma; ORR, objective response rate; OS, overall survival; PD-L1, programmed death-ligand 1; PFS, progression-free survival; SH-HCC, steatohepatitic hepatocellular carcinoma; SQ-HCC, scirrhous hepatocellular carcinoma; TMAs, tissue microarrays; TPS, tumor proportion score; VEGFA, vascular endothelial growth factor A; VETC, vessels encapsulating tumor clusters; VEGFR, Vascular Endothelial Growth Factor Receptor; EGFR, Epithelial growth factor receptor; PDGFR, platelet derived growth factor receptor; ICI, immune checkpoint inhibitors; TKI, tyrosine kinase inhibitors; TACE, transarterial chemoembolization; TARE, transarterial radioembolization.

Financial support

The research leading to these results has received funding from Associazione Italiana per la Ricerca sul Cancro (AIRC) under IG 2020 - ID. 25087 project – P.I. LDT.

Conflicts of interest

OR received honoraria for lectures and support for attending meetings or travel from Roche. LR reports grant/institutional research funding from AbbVie, AstraZeneca, BeiGene, Exelixis, Fibrogen, Incyte, Ipsen, Jazz Pharmaceuticals, MSD, Nerviano Medical Sciences, Roche, Servier, Taiho Oncology, TransThera Sciences, Zymeworks; consulting fees from AbbVie, AstraZeneca, Basilea, Bayer, Boehringer Ingelheim, Bristol Myers Squibb, Eisai, Elevar Therapeutics, Exelixis, Genenta, Guerbet, Hengrui, Incyte, Ipsen, Jazz Pharmaceuticals, MSD, Nerviano Medical Sciences, Roche, Servier, Taiho Oncology, Zymeworks; lecture fees from AstraZeneca, Bayer, Biologix, Bristol Myers Squibb, Eisai, Guerbet, Incyte, Ipsen, Roche, Servier; travel expenses from AstraZeneca, Servier.

Please refer to the accompanying ICMJE disclosure forms for further details.

Authors' contributions

Study concept and design: AS, VP, AB. Acquisition of data: AS, CC, ALB, MZ, LDT, AB. Analysis and interpretation of data: AS, VP, AB, MA. Drafting of the manuscript: AS, VP, AB. Critical revision of the manuscript for important intellectual content: VP, AB, CC, ALB, BT, JC, FL, CG, MD, LL, LM, GA, AP, OR, FM, MDB, MB, EB, MZ, LDT, LR, JCN. Statistical analysis: AS, EB. Obtained funding: AB. Study supervision: AB, VP, MB, MDB.

Data availability

The datasets used and/or analyzed during the current study are available from the corresponding author on reasonable request.

Supplementary data

Supplementary data to this article can be found online at https://doi.org/10.1016/j.jhepr.2026.101874.

References

Author names in bold designate shared co-first authorship

[1] Bray F, Laversanne M, Sung H, et al. Global cancer statistics 2022: GLOBOCAN estimates of incidence and mortality worldwide for 36 cancers in 185 countries. CA A Cancer J Clin 2024;74:229–263.

[2] Tran S, Zou B, Kam L, et al. Updates in characteristics and survival rates of hepatocellular carcinoma in a nationwide cohort of real-world US patients, 2003–2021. J Hepatocell Carcinoma 2023;10:2147–2158.

[3] Calderon-Martinez E, Landazuri-Navas S, Vilchez E, et al. Prognostic scores and survival rates by etiology of hepatocellular carcinoma: a review. J Clin Med Res 2023;15:200–207.

[4] Finn RS, Qin S, Ikeda M, et al. Atezolizumab plus bevacizumab in unresectable hepatocellular carcinoma. N Engl J Med 2020;382:1894–1905.

[5] Cheng AL, Qin S, Ikeda M, et al. Updated efficacy and safety data from IMbrave150: atezolizumab plus bevacizumab vs. sorafenib for unresectable hepatocellular carcinoma. J Hepatol 2022;76:862–873.

[6] Vogel A, Chan SL, Dawson LA, et al. Hepatocellular carcinoma: ESMO Clinical Practice Guideline for diagnosis, treatment and follow-up. Ann Oncol 2025;36:491–506.

[7] Sangro B, Argemi J, Ronot M, et al. EASL Clinical Practice Guidelines on the management of hepatocellular carcinoma. J Hepatol 2025;82:315–374.

[8] Abou-Alfa GK, Lau G, Kudo M, et al. Tremelimumab plus durvalumab in unresectable hepatocellular carcinoma. NEJM Evid 2022;1. EVIDoa210007070.

[9] Sangro B, Chan SL, Kelley RK, et al. Four-year overall survival update from the phase III HIMALAYA study of tremelimumab plus durvalumab in unresectable hepatocellular carcinoma. Ann Oncol 2024;35:448–457.

[10] **Rimassa L, Chan SL**, Sangro B, et al. Five-year overall survival update from the HIMALAYA study of tremelimumab plus durvalumab in unresectable HCC. J Hepatol 2025;83:899–908.

[11] **Yau T**, **Galle PR**, Decaens T, et al. Nivolumab plus ipilimumab versus lenvatinib or sorafenib as first-line treatment for unresectable hepatocellular carcinoma (CheckMate 9DW): an open-label, randomised, phase 3 trial. Lancet 2025;405:1851–1864.
[12] **Yang H**, **Kang B**, **Ha Y**, **Lee SH**, et al. High serum IL-6 correlates with reduced clinical benefit of atezolizumab and bevacizumab in unresectable hepatocellular carcinoma. JHEP Rep 2023;5:100672.
[13] Ueno M, Kosaka H, Iida H, et al. Response prediction model of atezolizumab plus bevacizumab in patients with unresectable hepatocellular carcinoma: multicenter retrospective study. Oncology 2023;101:565–574.
[14] Scheiner B, Pomej K, Kirstein MM, et al. Prognosis of patients with hepatocellular carcinoma treated with immunotherapy – development and validation of the CRAFITY score. J Hepatol 2022;76:353–363.
[15] Zhu AX, Abbas AR, De Galarreta MR, et al. Molecular correlates of clinical response and resistance to atezolizumab in combination with bevacizumab in advanced hepatocellular carcinoma. Nat Med 2022;28:1599–1611.
[16] Zeng Q, Klein C, Caruso S, et al. Artificial intelligence-based pathology as a biomarker of sensitivity to atezolizumab–bevacizumab in patients with hepatocellular carcinoma: a multicentre retrospective study. Lancet Oncol 2023;24:1411–1422.
[17] **Cappuyns S**, **Piqué-Gili M**, **Esteban-Fabró R**, et al. Single-cell RNA sequencing-derived signatures define response patterns to atezolizumab + bevacizumab in advanced hepatocellular carcinoma. J Hepatol 2025;82:1036–1049.
[18] **Renne SL**, **Woo HY**, **Allegra S**, et al. Vessels encapsulating tumor clusters (VETC) is a powerful predictor of aggressive hepatocellular carcinoma. Hepatology 2020;71:183–195.
[19] **Wang J**, **Li X**, **Tang H**, et al. Vessels that encapsulate tumor clusters (VETC) pattern predicts the efficacy of adjuvant TACE in hepatocellular carcinoma. J Cancer Res Clin Oncol 2023;149:4163–4172.
[20] Fang J, Xu L, Shang L, et al. Vessels that encapsulate tumor clusters (VETC) pattern is a predictor of sorafenib benefit in patients with hepatocellular carcinoma. Hepatology 2019;70:824–839.
[21] Liu K, Dennis C, Prince DS, et al. Vessels that encapsulate tumour clusters vascular pattern in hepatocellular carcinoma. JHEP Rep 2023;5:100792.
[22] **Guo B**, **Cai G**, **Zhu J**, **Chen Q**, **Li D**, et al. Vessels encapsulating tumor clusters is associated with impaired efficacy of adjuvant immunotherapy in resected hepatocellular carcinoma. J Adv Res 2025;23. https://doi.org/10.1016/j.jare.2025.11.054. S2090-1232(25)00950-6.
[23] Huang BY, Mi ZQ, Zhang XY, et al. Vessels encapsulating tumor clusters promote noninvasive metastasis of hepatocellular carcinoma by shaping an immunosuppressive microenvironment. J Clin Invest 2026;136:e193758.
[24] De Carlo C, Rosman-Nathanson R, Durante B, et al. The tumor microenvironment of VETC+ hepatocellular carcinoma is enriched of immunosuppressive TAMs spatially close to endothelial cells. Dig Liver Dis 2025;57:62–67.
[25] Zhou HC, Liu CX, Pan WD, et al. Dual and opposing roles of the androgen receptor in VETC-dependent and invasion-dependent metastasis of hepatocellular carcinoma. J Hepatol 2021;75:900–911.
[26] De Carlo C, Akpinar R, Durante B, et al. Vessels that encapsulate tumor cluster+ unresectable hepatocellular carcinomas benefit of antiangiogenic therapy. Liver Cancer 2025. https://doi.org/10.1159/00054766.
[27] Lencioni R, Llovet J. Modified RECIST (mRECIST) assessment for hepatocellular carcinoma. Semin Liver Dis 2010;30:52–60.
[28] WHO classification of Tumours Editorial Board. Digestive system tumours. 5th ed, 1. Lyon: International Agency for Research on cancer; 2019.
[29] Akiba J, Nakayama M, Sadashima E, et al. Prognostic impact of vessels encapsulating tumor clusters and macrotrabecular patterns in hepatocellular carcinoma. Pathol Res Pract 2022;238:154084.
[30] Fleiss JL, Cohen J. The equivalence of weighted kappa and the intraclass correlation coefficient as measures of reliability. Educ Psychol Meas 1973;33:613–619.
[31] Cicchetti DV, Allison T. A new procedure for assessing reliability of scoring EEG sleep recordings. Am J EEG Technol 1971;11:101–110.
[32] Ballman KV. Biomarker: predictive or prognostic? J Clin Oncol 2015;33:3968–3971.
[33] Zhang P, Ono A, Fujii Y, et al. The presence of vessels encapsulating tumor clusters is associated with an immunosuppressive tumor microenvironment in hepatocellular carcinoma. Int J Cancer 2022;151:2278–2290.
[34] Kurebayashi Y, Matsuda K, Ueno A, et al. Immunovascular classification of HCC reflects reciprocal interaction between immune and angiogenic tumor microenvironments. Hepatology 2022;75:1139–1153.
[35] Itoh S, Yoshizumi T, Yugawa K, et al. Impact of immune response on outcomes in hepatocellular carcinoma: association with vascular formation. Hepatology 2020;72:1987–1999.
[36] Ding T, Xu J, Zhang Y, et al. Endothelium-coated tumor clusters are associated with poor prognosis and micrometastasis of hepatocellular carcinoma after resection. Cancer 2011;117:4878–4889.
[37] Calderaro J, Couchy G, Imbeaud S, et al. Histological subtypes of hepatocellular carcinoma are related to gene mutations and molecular tumour classification. J Hepatol 2017;67:727–738.
[38] Trapani L, Beaufrère A, Hobeika C, et al. Pathological overview of steatohepatitic hepatocellular carcinoma in a surgical series. Histopathology 2023;83:526–537.
[39] Jeon Y, Benedict M, Taddei T, et al. Macrotrabecular Hepatocellular carcinoma: an aggressive subtype of hepatocellular carcinoma. Am J Surg Pathol 2019;43:943–948.
[40] **Brusset B**, **Jacquemin M**, Teyssier Y, et al. Radiological diagnosis of hepatocellular carcinoma does not preclude biopsy before treatment. JHEP Rep 2024;6:100957.
[41] Beaufrère A, Caruso S, Calderaro J, et al. Gene expression signature as a surrogate marker of microvascular invasion on routine hepatocellular carcinoma biopsies. J Hepatol 2022;76:343–352.

Keywords: Vessels encapsulating tumor clusters; Hepatocellular carcinoma; Atezolizumab; Bevacizumab; Histology; Response.
Received 28 February 2026; received in revised form 12 April 2026; accepted 17 April 2026; Available online 29 April 2026

Journal of Hepatology, Volume *8*

Supplemental information

Vessels encapsulating tumor clusters predict better outcomes in advanced hepatocellular carcinoma treated with atezolizumab–bevacizumab

Alexandre Sayadi, Claudia Campani, Astrid Laurent-Bellue, Marianne Ziol, Luca Di Tommaso, Etienne Becht, Benoit Terris, Julien Calderaro, Luca Messerini, Miguel Albuquerque, Federico Lipsich, Carolina Gutierrez, Marion Dhooge, Lisa Lellouche, Giuliana Amaddeo, Alina Pascale, Olivier Rosmorduc, Fabio Marra, Lorenza Rimassa, Marco Dioguardi Burgio, Jean-Charles Nault, Mohamed Bouattour, Valérie Paradis, and Aurélie Beaufrère

Vessels encapsulating tumor clusters predict better outcomes in advanced hepatocellular carcinoma treated with atezolizumab–bevacizumab

Alexandre Sayadi, Claudia Campani, Astrid Laurent-Bellue, Marianne Ziol, Luca Di Tommaso, Etienne Becht, Benoit Terris, Julien Calderaro, Luca Messerini, Miguel Albuquerque, Federico Lipsich, Carolina Gutierrez, Marion Dhooge, Lisa Lellouche, Giuliana Amaddeo, Alina Pascale, Olivier Rosmorduc, Fabio Marra, Lorenza Rimassa, Marco Dioguardi Burgio, Jean-Charles Nault, Mohamed Bouattour, Valérie Paradis, Aurélie Beaufrère

Table of contents

Supplementary methods 3
Supplementary references 4
Table S1 5
Table S2 6
Table S3 7
Table S4 7
Table S5 8
Table S6 10
Table S7 11
Table S8 13
Table S9 15
Table S10 17

Table S11 19

Fig. S1. 22

Fig. S2 23

Fig. S3 24

Fig. S4. 25

Fig. S5. 26

Fig. S6 27

Fig. S7 28

Fig. S8. 29

Fig. S9 30

Supplementary methods

Clinical data collection

The following baseline characteristics were collected: age, sex, height, weight, underlying liver disease etiology (HCV and/or HBV infection, Metabolic-dysfunction Associated Steatotic Liver Disease [MASLD], Alcohol-associated Liver Disease [ALD], Metabolic and Alcohol-associated Liver Disease [MetALD]), Eastern Cooperative Oncology Group (ECOG) Performance Status, presence of underlying cirrhosis and Child-Pugh class[1]. Tumor characteristics included macrovascular invasion, extrahepatic spread and BCLC stage. Prior locoregional or systemic treatment were also recorded. Baseline blood tests included bilirubin, international normalized ratio (INR), C-reactive protein (CRP), and alpha-fetoprotein (AFP) levels.

Immunohistochemistry

For Beaujon Hospital's cases in the discovery cohort, supplementary FFPE tissue sections were stained with the following antibodies, depending on remaining material: CD3 (DAKO, A0452, 1:100), CD8 (DAKO, C8/144B, M7103, 1:50), PD-L1 (QUARTETT, QR1, 1-PR292-02, 1:100), glutamine synthetase (GS, Chemicon, GS-6, MAB302, 1:200), β-catenin (BD Biosciences, 14/Beta-Catenin, 610153, 1:200) and p53 (DAKO, DO7, M700101, 1:100). Intratumoral CD8+ and CD3+ T cell density was determined as the density per mm² of CD8-stained cells occupying the tumor, using positive cell detection in QuPath software (v.0.5.0)[2].

Tumor proportion score (TPS) was defined as the number of PD-L1+ tumor cells divided by all tumor cells; whereas Combined Positive Score (CPS) was defined as the

number of PD-L1+ tumor cells and inflammatory cells (i.e., lymphocytes and macrophages) divided by all tumor cells. *CTNNB1* mutational status was inferred from diffuse and intense GS staining and/or nuclear β-catenin localization. *TP53* mutational status was inferred from strong, diffuse nuclear p53 staining or complete absence of staining.

Supplementary references

[1] Rinella ME, Lazarus JV, Ratziu V, et al. A multisociety Delphi consensus statement on new fatty liver disease nomenclature. Journal of Hepatology 79:1542-56.

[2] Bankhead P, Loughrey MB, Fernández JA, et al. QuPath: Open source software for digital pathology image analysis. Sci Rep 7. Available on: https://www.nature.com/articles/s41598-017-17204-5.

Table S1. Criteria for histopathological assessment

Criteria	Assessment
Histopathologic subtype (according to WHO)	**Steatohepatitic (SH):** Among the 5 following criteria (ballooning, Mallory's body, inflammation, fibrosis and steatosis); presence of at least 3 criteria on more than 50% of the tumor surface area
	**Macrotrabecular massive (MTM):** Macrotrabecular architecture (hepatic cord thickness >6 cells) over more than 50% of tumor surface area
	**Scirrhous (SQ):** Abundant fibrosis on more than 50% of the tumor surface, without criteria for SH-HCC
	**Clear cell (CC):** Tumor cells clarified on more than 80% of tumor surface area
	**Neutrophil-rich (NR):** Neutrophil-rich infiltrate covering more than 50% of the tumor surface, with no other predominant subtype
	**Lymphocyte-rich (LR):** Lymphocyte-rich infiltrate over 50% of tumor surface, with no other predominant subtype
	**Not Other Specified (NOS):** Exclusion diagnosis after elimination of subtypes listed above
VETC phenotype	- Non-VETC-HCC: No VETC images<br>- Incomplete VETC phenotype: Focal VETC images (1 - 54% of tumor surface)<br>- Complete VETC phenotype: Diffuse VETC images (i.e., « VETC-HCC », ≥55% of tumor surface)
Tumoral differentiation (WHO)	- I : Well differentiated<br>- II : Moderately differentiated<br>- III : Poorly differentiated
Tumoral necrosis	Percentage of tumoral necrosis (0 to 100).
Tumoral fibrosis	Percentage of tumoral fibrosis (0 to 100).
Tumoral inflammatory infiltrate	Semi-quantitative (Absent, Low, Moderate, High).
Tumoral steatosis	Percentage of steatotic tumoral cells (0 to 100).
Tumoral cholestasis	Absent / Present

Table S2. Correlation between histopathological criteria and response in the discovery cohort

**Characteristic**	**No response** N = 122[1]	**Response** N = 86[1]	**p-value**[2]
Macrotrabecular massive-HCC	39 (32%)	19 (22%)	0.12
Steatohepatitic-HCC	15 (12%)	9 (10%)	0.7
Squirrhous-HCC	10 (8.2%)	6 (7.0%)	0.7
Clear cell-HCC	7 (5.7%)	2 (2.3%)	0.3
Not otherwise specified-HCC	50 (41%)	48 (56%)	**0.035**
Tumoral necrosis	33 (27%)	25 (29%)	0.7
Tumoral inflammatory infiltrate			0.5
Absent	55 (45%)	36 (42%)	
Low	35 (29%)	28 (33%)	
Intermediate	22 (18%)	11 (13%)	
High	10 (8.2%)	11 (13%)	
Tumoral steatosis	29 (24%)	23 (27%)	0.6
Tumoral cholestasis	10 (8.2%)	10 (12%)	0.4
Differentiation (WHO)			0.7
I	44 (36%)	36 (42%)	
II	62 (51%)	40 (47%)	
III	16 (13%)	10 (12%)	
VETC phenotype			**0.002**
Non or Incomplete VETC-HCC	111 (91%)	65 (76%)	
VETC-HCC	11 (9.0%)	21 (24%)	

[1]n (%); [2]Pearson's Chi-squared test

Table S3. Contingency table between HES and CD34 assessment for the VETC phenotype in the discovery cohort

	No VETC (CD34)	Incomplete VETC-HCC (CD34)	VETC-HCC (CD34)	Total
No VETC (HES)	115	5	1	121
Incomplete VETC-HCC (HES)	1	18	3	22
VETC-HCC (HES)	0	0	19	19
Total	116	23	23	162

Table S4. Agreement between CD34 and HES staining for VETC phenotype in the discovery cohort

Agreement metric	Kappa	Lower 95% CI	Upper 95% CI	ASE	z-score	p-value
Unweighted kappa	0.856	0.772	0.940	0.043	19.907	<0.001
Weighted kappa (linear)	0.890	0.821	0.959	0.035	25.429	<0.001

Table S5. Characteristics of HCC with VETC images in the discovery cohort

Characteristic	Non-VETC-HCC N = 151[1]	Incomplete and Complete VETC-HCC N = 57[1]	p-value[2]
Viral hepatitis (HBV/HCV)	66 (44%)	27 (47%)	0.6
Child Pugh class			0.2
No cirrhosis	50 (33%)	26 (46%)	
A	82 (54%)	23 (40%)	
B	19 (13%)	8 (14%)	
Macrovascular invasion	65 (43%)	22 (39%)	0.6
Extrahepatic spread	53 (35%)	21 (37%)	0.8
AFP[3]	49.5 (6.0-5,077.0)	133.0 (6.4-3,826.0)	0.8
CRP[4]	11.5 (4.0-31.0)	11.0 (6.0-60.0)	0.8
CRAFITY[5]			0.5
low	35 (29%)	13 (29%)	
intermediate	51 (42%)	15 (33%)	
high	35 (29%)	17 (38%)	
Macrotrabecular massive-HCC	37 (25%)	21 (37%)	0.077
Steatohepatitic-HCC	23 (15%)	1 (1.8%)	0.007
Squirrhous-HCC	15 (9.9%)	1 (1.8%)	0.076
Clear cell-HCC	7 (4.6%)	2 (3.5%)	0.9
Not otherwise specified-HCC	66 (44%)	32 (56%)	0.11
p53 expression[6]	25 (34%)	9 (28%)	0.6
PD-L1 > 0 (TPS and/or CPS)[7]	24 (42%)	9 (31%)	0.3
B-catenin expression[8]	14 (17%)	14 (41%)	0.005
CD8+ T cells (/mm²)[6]	74.2 (29.2-237.4)	45.6 (27.9-128.7)	0.066
CD3+ T cells (/mm²)[9]	285.5 (112.0-557.0)	157.1 (92.9-335.4)	0.040
Objective Response Rate			0.086

Response	57 (38%)	29 (51%)

[1]Median (Q1 - Q3); n (%);[2]Wilcoxon rank sum test; Pearson's Chi-squared test [3]Available for 205 cases; [4]Available for 169 cases; [5]Available for 166 cases; [6]Available for 106 cases; [7]Available for 86 cases; [8]Available for 117 cases; [9]Available for 100 cases

Table S6. Sensitivity Analysis of VETC Thresholds in patients treated with atezo+bev using PFS (Discovery Cohort)

Threshold (%)	HR	Lower 95% CI	Upper 95% CI	p-value	n patients > threshold	n patients < threshold	Proportion patients > threshold
**55**	**0.528**	**0.336**	**0.828**	**0.005**	**32**	**176**	**0.154**
**50**	**0.618**	**0.411**	**0.929**	**0.021**	**38**	**170**	**0.183**
**60**	**0.615**	**0.389**	**0.972**	**0.037**	**29**	**179**	**0.139**
40	0.710	0.491	1.026	0.068	49	159	0.236
45	0.716	0.486	1.054	0.090	42	166	0.202
65	0.742	0.455	1.208	0.230	24	184	0.115
35	0.811	0.571	1.152	0.242	54	154	0.260
70	0.780	0.466	1.306	0.345	21	187	0.101
90	0.705	0.331	1.503	0.365	10	198	0.048
30	0.869	0.619	1.221	0.418	58	150	0.279
75	0.821	0.456	1.476	0.509	16	192	0.077
15	0.901	0.658	1.234	0.516	72	136	0.346
25	0.924	0.667	1.280	0.635	64	144	0.308
85	0.873	0.461	1.653	0.676	13	195	0.062
20	0.940	0.683	1.292	0.701	69	139	0.332
10	0.963	0.708	1.311	0.810	78	130	0.375
5	0.964	0.710	1.309	0.816	80	128	0.385
80	0.932	0.506	1.718	0.822	14	194	0.067
95	0.967	0.308	3.034	0.954	4	204	0.019

Table S7. Clinical and biological characteristics of *TP53* and *CTNNB1* mutated HCC in patients from Beaujon hospital in the discovery cohort

**Characteristic**	***CTNNB1*-mutated expression** N = 25[1]	***TP53*-mutated expression** N = 31[1]	**Double positive** N = 3[1]	**Double negative** N = 46[1]	**p-value**[2]
AFP (ng/mL)[3]	38.0 (4.0-2,542.5)	849.0 (45.0-14,300.0)	11.0 (4.0-316,831.0)	61.0 (5.4-5,215.0)	0.4
CRAFITY score[4]					0.3
low	6 (26%)	3 (10%)	0 (0%)	13 (31%)	
intermediate	11 (48%)	12 (41%)	2 (67%)	14 (33%)	
high	6 (26%)	14 (48%)	1 (33%)	15 (36%)	
Viral etiology	9 (36%)	18 (58%)	3 (100%)	20 (43%)	0.10
Differentiation (WHO)					<0.001
I	15 (60%)	2 (6.5%)	0 (0%)	17 (37%)	
II	9 (36%)	18 (58%)	3 (100%)	23 (50%)	
III	1 (4.0%)	11 (35%)	0 (0%)	6 (13%)	
BCLC stage					0.2
B	11 (44%)	6 (19%)	1 (33%)	12 (26%)	
C	14 (56%)	25 (81%)	2 (67%)	34 (74%)	
Histological subtype					
NOS	22 (88%)	7 (23%)	0 (0%)	22 (48%)	
CC	0 (0%)	2 (6.5%)	0 (0%)	1 (2.2%)	
MTM	2 (8.0%)	17 (55%)	3 (100%)	9 (20%)	
SH	1 (4.0%)	1 (3.2%)	0 (0%)	7 (15%)	
SQ	0 (0%)	4 (13%)	0 (0%)	7 (15%)	
VETC					0.6
Non or Incomplete VETC-HCC	21 (84%)	28 (90%)	2 (67%)	40 (87%)	
VETC-HCC	4 (16%)	3 (9.7%)	1 (33%)	6 (13%)	
CD8+ T cells density[5]	38.4 (22.2-92.1)	136.2 (42.4-371.1)	5.3 (0.0-52.4)	61.9 (33.6-141.7)	0.013

Proliferation index (Ki67, %)[6]	5.0 (3.6-7.8)	28.7 (19.6-34.9)	25.0 (5.0-33.3)	10.7 (4.1-16.5)	<0.001
TPS > 1%[7]					0.2
0	15 (94%)	17 (68%)	3 (100%)	29 (83%)	
1	1 (6.3%)	8 (32%)	0 (0%)	6 (17%)	
Objective Response Rate					0.9
No response	16 (64%)	19 (61%)	2 (67%)	27 (59%)	
Response	9 (36%)	12 (39%)	1 (33%)	19 (41%)	

[1]Median (IQR); n (%); [2]Kruskal-Wallis rank sum test; Fisher's exact test; [3]Available for 205 cases; [4]Available for 164 cases; [5]Available for 106 cases; [6]Available for 95 cases; [7]Available for 86 cases

Table S8. Univariable and multivariable Cox regression analyses of prognostic factors for progression-free and overall survival in the discovery cohort.

		Univariable (PFS)			Multivariable (PFS)				Univariable (OS)			Multivariable (OS)		
**Characteristic**	**N**	**HR**	**95% CI**	**p-value**	**HR**	**95% CI**	**p-value**	**N**	**HR**	**95% CI**	**p-value**	**HR**	**95% CI**	**p-value**
Sex	208							208						
Male		—	—		—	—			—	—		—	—	
Female		0.82	0.52, 1.28	0.4	0.78	0.49, 1.25	0.3		0.98	0.59, 1.61	0.9	0.95	0.57, 1.61	0.9
Age	208							208						
<65 years		—	—		—	—			—	—		—	—	
>65 years		0.96	0.70, 1.32	0.8	1.11	0.75, 1.63	0.6		0.95	0.66, 1.37	0.8	1.06	0.70, 1.63	0.8
ECOG PS	208							208						
0		—	—		—	—			—	—		—	—	
≥ 1		1.33	0.98, 1.79	0.064	1.37	0.98, 1.93	0.066		1.74	1.23, 2.46	0.002	1.69	1.14, 2.50	0.008
Child Pugh	208							208						
No cirrhosis		—	—		—	—			—	—		—	—	
A		0.77	0.56, 1.06	0.10	0.74	0.52, 1.05	0.089		0.67	0.46, 0.98	0.037	0.72	0.48, 1.06	0.10
B		1.56	0.98, 2.47	0.061	1.80	1.04, 3.12	0.035		2.28	1.38, 3.79	0.001	2.84	1.54, 5.22	<0.001
Viral etiology	208							208						
Yes		—	—		—	—			—	—		—	—	
No		1.13	0.84, 1.52	0.4	1.30	0.90, 1.87	0.2		1.22	0.87, 1.73	0.3	1.45	0.97, 2.18	0.071
BCLC	208							208						
B		—	—		—	—			—	—		—	—	
C		1.01	0.74, 1.38	>0.9	1.09	0.77, 1.54	0.6		1.04	0.73, 1.49	0.8	1.05	0.70, 1.58	0.8
AFP > 400 $ng.mL^{-1}$	205							205						
No		—	—		—	—			—	—		—	—	
Yes		1.56	1.15, 2.11	0.004	1.78	1.28, 2.46	<0.001		1.45	1.02, 2.05	0.036	1.78	1.22, 2.59	0.003
Previous treatment	208							208						
No		—	—		—	—			—	—		—	—	
Yes		0.88	0.62, 1.25	0.5	0.98	0.67, 1.42	0.9		0.75	0.49, 1.14	0.2	0.87	0.56, 1.37	0.6
Center	208							208						
Beaujon		—	—						—	—				
Other		0.82	0.59, 1.14	0.2					0.86	0.59, 1.25	0.4			

Histological subtype	208							208						
NOS		—	—		—	—			—	—		—	—	
CC		1.82	0.91, 3.63	0.090	2.26	1.07, 4.77	0.032		1.63	0.78, 3.42	0.2	1.68	0.76, 3.70	0.2
MTM		1.35	0.95, 1.92	0.10	1.37	0.94, 2.00	0.10		1.36	0.90, 2.05	0.15	1.32	0.85, 2.06	0.2
SH		1.07	0.66, 1.72	0.8	1.07	0.55, 2.06	0.8		1.28	0.74, 2.24	0.4	1.51	0.68, 3.35	0.3
SQ		1.77	1.04, 3.04	0.037	1.57	0.90, 2.76	0.11		1.87	1.02, 3.42	0.041	1.60	0.84, 3.05	0.15
VETC	208							208						
Non- or Incomplete-VETC-HCC		—	—		—	—			—	—		—	—	
VETC-HCC		0.53	0.34, 0.83	0.005	0.42	0.26, 0.68	<0.001		0.53	0.31, 0.91	0.022	0.46	0.26, 0.82	0.009
Differentiation	208							208						
Well		—	—						—	—				
Moderately		1.09	0.79, 1.49	0.6					1.12	0.78, 1.62	0.5			
Poorly		1.17	0.72, 1.89	0.5					1.10	0.64, 1.88	0.7			
Inflammatory infiltrate	208							208						
Absent		—	—						—	—				
Low		0.96	0.68, 1.36	0.8					1.08	0.72, 1.62	0.7			
Intermediate		0.89	0.58, 1.37	0.6					1.25	0.78, 2.03	0.4			
High		1.09	0.66, 1.83	0.7					0.99	0.53, 1.85	>0.9			
Tumoral necrosis	208							208						
No		—	—						—	—				
Yes		1.20	0.86, 1.66	0.3					1.05	0.72, 1.54	0.8			
Tumoral steatosis	208							208						
No		—	—		—	—			—	—		—	—	
Yes		0.76	0.54, 1.07	0.11	0.80	0.50, 1.28	0.4		0.70	0.47, 1.05	0.081	0.61	0.34, 1.09	0.10
Tumoral cholestasis	208							208						
No		—	—						—	—				
Yes		0.84	0.50, 1.41	0.5					1.07	0.61, 1.91	0.8			

Abbreviations: CI = Confidence Interval, HR = Hazard Ratio

Table S9. Progression-Free and Overall Survival Cox model including treatment interaction (VETC x treatment) in the validation cohort.

	PFS			OS		
**Characteristic**	**HR**	**95% CI**	**p-value**	**HR**	**95% CI**	**p-value**
AFP > 400 ng/mL vs AFP < 400 ng/mL	1.17	0.76, 1.79	0.5	1.47	0.93, 2.32	0.10
ECOG PS ≥ 1 vs 0	1.28	0.84, 1.95	0.3	1.66	1.02, 2.68	0.040
BCLC C vs B	1.19	0.75, 1.90	0.5	1.66	0.98, 2.81	0.058
Age ≥ 65 vs <65 years old	0.71	0.47, 1.07	0.10	0.66	0.40, 1.07	0.088
Female vs Male	1.14	0.68, 1.93	0.6	0.94	0.51, 1.76	0.9
Previous locoregional treatment vs none	1.21	0.74, 1.96	0.5	1.36	0.81, 2.29	0.2
Child Pugh class						
No cirrhosis	—	—		—	—	
A	0.70	0.45, 1.10	0.13	1.04	0.63, 1.71	0.9
B	0.96	0.45, 2.02	0.9	1.52	0.68, 3.38	0.3
Etiology : Non-viral vs viral	1.20	0.76, 1.88	0.4	1.80	1.09, 2.95	0.021
**Interaction term : VETC status X treatment**	0.28	0.10, 0.79	0.016	0.15	0.05, 0.48	0.001
**Adjusted subgroup hazard ratios**						
VETC-HCC vs non-VETC-HCC in patients treated with durva+treme	2.44	1.12–5.29	0.024	3.90	1.66–9.15	0.002
VETC-HCC vs non-VETC-HCC in patients treated with atezo+bev	0.69	0.35–1.36	0.280	0.61	0.29–1.3	0.2
Atezo+bev vs durva+treme in patients with VETC-HCC	0.43	0.17–1.06	0.067	0.19	0.07–0.51	0.001
Atezo+bev vs durva+treme in	1.52	0.93–2.48	0.095	1.22	0.69–2.13	0.5

patients with non-VETC-HCC	

Abbreviations: CI = Confidence Interval, HR = Hazard Ratio

Table S10. Progression-Free and Overall Survival Cox model including treatment interaction (VETC x treatment) in the whole cohort (discovery + validation).

	PFS			OS		
**Characteristic**	**HR**	**95% CI**	**p-value**	**HR**	**95% CI**	**p-value**
AFP ≥ 400 ng/mL vs AFP < 400 ng/mL	1.47	1.15, 1.88	0.002	1.56	1.18, 2.06	0.002
ECOG PS ≥ 1 vs 0	1.32	1.03, 1.69	0.029	1.76	1.33, 2.33	<0.001
BCLC C vs B	1.14	0.87, 1.48	0.3	1.26	0.93, 1.70	0.13
Age ≥ 65 vs <65 years old	0.89	0.67, 1.17	0.4	0.86	0.63, 1.18	0.3
Female vs Male	0.95	0.68, 1.32	0.7	0.97	0.66, 1.42	0.9
Previous locoregional treatment vs none	1.16	0.89, 1.53	0.3	1.22	0.90, 1.67	0.2
Child Pugh Score						
No cirrhosis	—	—		—	—	
A	0.71	0.54, 0.92	0.011	0.76	0.57, 1.03	0.081
B	1.25	0.83, 1.88	0.3	1.94	1.23, 3.04	0.004
Etiology : Non-viral vs viral	1.17	0.89, 1.54	0.3	1.46	1.07, 1.98	0.016
**Interaction term : VETC status X treatment**	0.22	0.10, 0.51	<0.001	0.15	0.06, 0.38	<0.001
**Adjusted subgroup hazard ratios**						
VETC-HCC vs non-VETC-HCC in patients treated with durva+treme	2.50	1.18–5.29	0.017	3.54	1.56–8.03	0.002
VETC-HCC vs non-VETC-HCC in patients treated with atezo+bev	0.56	0.38–0.81	0.002	0.54	0.35–0.83	0.005
Atezo+bev vs durva+treme in patients with VETC-HCC	0.34	0.16–0.69	0.003	0.19	0.09–0.42	<0.001

Atezo+bev vs durva+treme in patients with non-VETC-HCC	1.51	0.98–2.31	0.06	1.26	0.77–2.07	0.3

Abbreviations: CI = Confidence Interval, HR = Hazard Ratio

Table S11. Sensitivity analyses of overall survival restricted to patients with OS ≥ 3 months.

Model	Term	HR	Lower 95% CI	Upper 95% CI	p-value
Discovery cohort (patients treated with Atezo+Bev) – OS ≥ 3 months	VETC-HCC (Yes vs No)	0.576	0.362	0.915	0.020
Discovery cohort (patients treated with Atezo+Bev) – OS ≥ 3 months	AFP > 400 ng/mL	1.660	1.201	2.295	0.002
Discovery cohort (patients treated with Atezo+Bev) – OS ≥ 3 months	Age > 65 years	1.131	0.773	1.654	0.526
Discovery cohort (patients treated with Atezo+Bev) – OS ≥ 3 months	BCLC C vs B	1.086	0.770	1.531	0.637
Discovery cohort (patients treated with Atezo+Bev) – OS ≥ 3 months	Child-Pugh A vs no cirrhosis	0.795	0.565	1.119	0.189
Discovery cohort (patients treated with Atezo+Bev) – OS ≥ 3 months	Child-Pugh B vs no cirrhosis	2.059	1.164	3.643	0.013
Discovery cohort (patients treated with Atezo+Bev) – OS ≥ 3 months	ECOG PS ≥ 1	1.601	1.152	2.227	0.005
Discovery cohort (patients treated with Atezo+Bev) – OS ≥ 3 months	Female vs male	0.902	0.575	1.414	0.653
Discovery cohort (patients treated with Atezo+Bev) – OS ≥ 3 months	Non-viral vs viral etiology	1.489	1.040	2.132	0.030
Discovery cohort (patients treated with Atezo+Bev) – OS ≥ 3 months	Previous locoregional treatment	1.312	0.923	1.867	0.131
Validation cohort – OS ≥ 3 months	VETC-HCC (Yes vs No) in patients treated with durva+treme	3.232	0.988	10.570	0.052
Validation cohort – OS ≥ 3 months	Interaction: VETC status x treatment	0.254	0.064	1.001	0.050

Validation cohort – OS ≥ 3 months	AFP > 400 ng/mL	1.383	0.827	2.312	0.217
Validation cohort – OS ≥ 3 months	Age > 65 years	0.875	0.497	1.540	0.643
Validation cohort – OS ≥ 3 months	BCLC C vs B	2.084	1.141	3.808	0.017
Validation cohort – OS ≥ 3 months	Child-Pugh A vs no cirrhosis	1.124	0.638	1.981	0.686
Validation cohort – OS ≥ 3 months	Child-Pugh B vs no cirrhosis	1.901	0.766	4.722	0.166
Validation cohort – OS ≥ 3 months	ECOG PS ≥ 1	1.249	0.740	2.106	0.405
Validation cohort – OS ≥ 3 months	Female vs male	0.692	0.335	1.429	0.320
Validation cohort – OS ≥ 3 months	Non-viral vs viral etiology	1.882	1.067	3.321	0.029
Validation cohort – OS ≥ 3 months	Previous locoregional treatment	1.498	0.839	2.673	0.172
Validation cohort – OS ≥ 3 months	Treatment: atezo+bev vs durva+treme	1.572	0.807	3.061	0.184
Whole cohort – OS ≥ 3 months	VETC-HCC (Yes vs No) in patients treated with durva+treme	3.310	1.045	10.488	0.042
Whole cohort – OS ≥ 3 months	Interaction: VETC status × treatment	0.174	0.051	0.598	0.005
Whole cohort – OS ≥ 3 months	AFP > 400 ng/mL	1.586	1.163	2.162	0.004
Whole cohort – OS ≥ 3 months	Age > 65 years	1.065	0.742	1.526	0.734
Whole cohort – OS ≥ 3 months	BCLC C vs B	1.154	0.832	1.601	0.389
Whole cohort – OS ≥ 3 months	Child-Pugh A vs no cirrhosis	0.801	0.576	1.114	0.188
Whole cohort – OS ≥ 3 months	Child-Pugh B vs no cirrhosis	2.080	1.211	3.571	0.008
Whole cohort – OS ≥ 3 months	ECOG PS ≥ 1	1.610	1.178	2.201	0.003

Whole cohort – OS ≥ 3 months	Female vs male	0.936	0.612	1.431	0.760
Whole cohort – OS ≥ 3 months	Non-viral vs viral etiology	1.467	1.042	2.064	0.028
Whole cohort – OS ≥ 3 months	Previous locoregional treatment	1.372	0.983	1.915	0.063
Whole cohort – OS ≥ 3 months	Treatment: atezo+bev vs durva+treme	1.530	0.842	2.782	0.163

Fig. S1. Flowchart.

A : Discovery Cohort

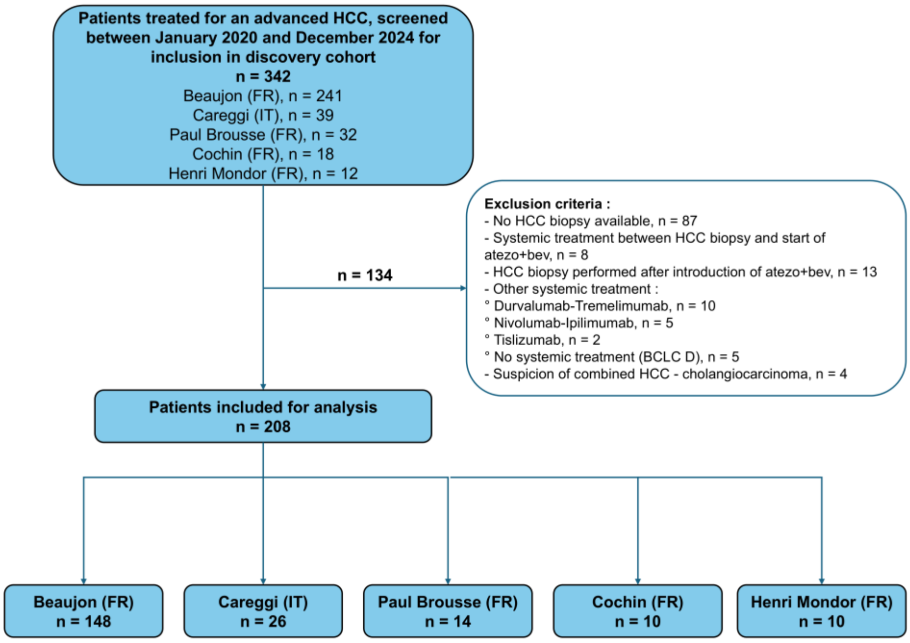

B : Validation cohort

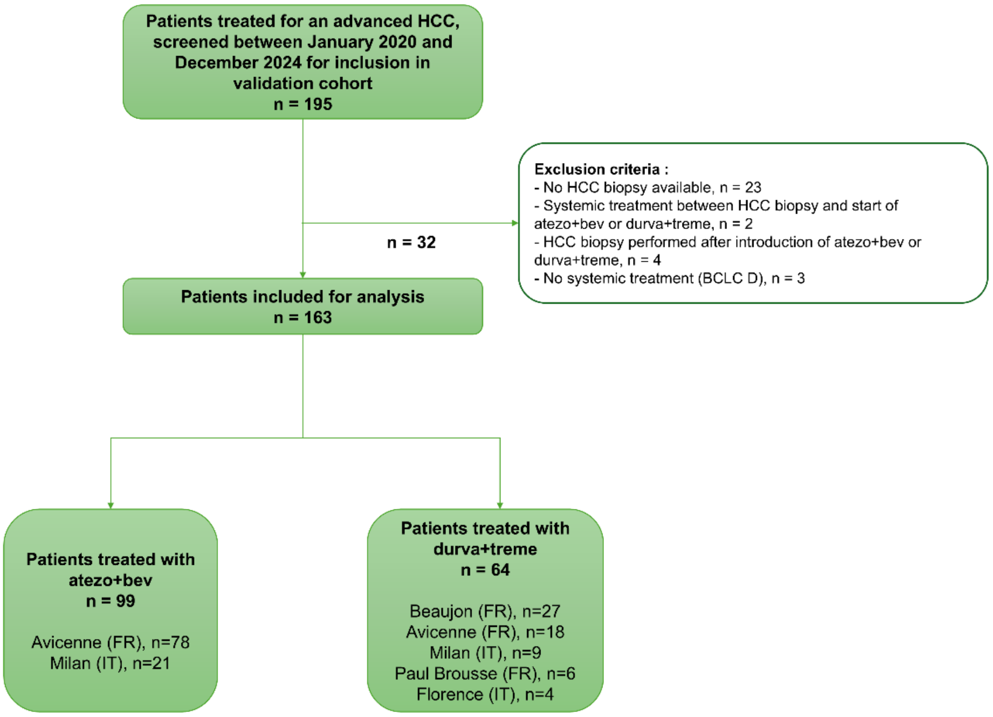

Fig. S2. Survival outcomes according to serum AFP and CRAFITY score in the discovery cohort

A, B: Progression-Free Survival and Overall Survival according to serum AFP (< 400 / > 400 ng.mL^{-1}). **C, D:** Progression-Free Survival and Overall Survival according to CRAFITY score (Low, Intermediate, High). HRs from univariate Cox regression are displayed with their 95% CI and p-value (Wald test).

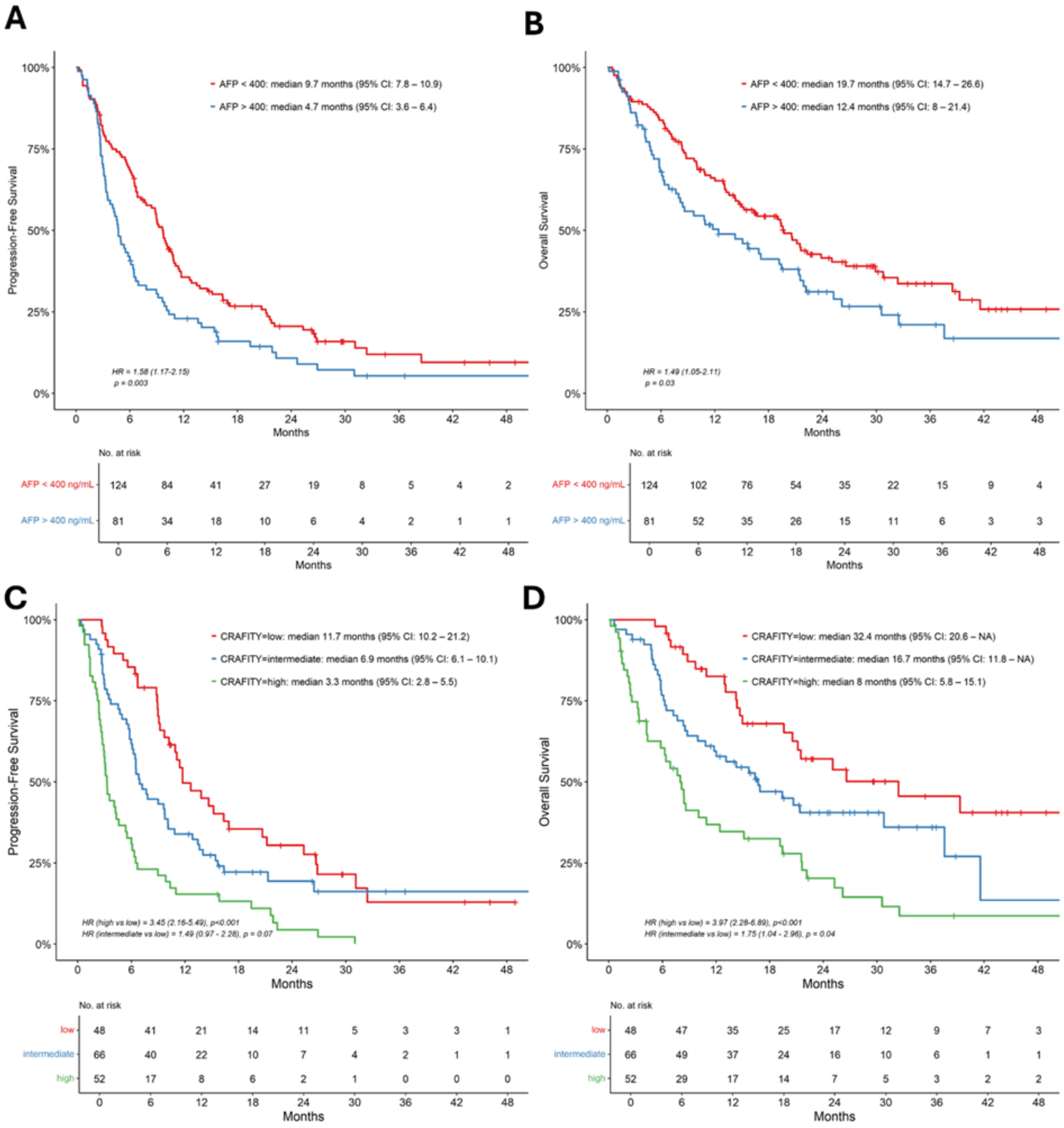

Fig. S3. Overall survival according to best response per mRECIST in the discovery cohort.

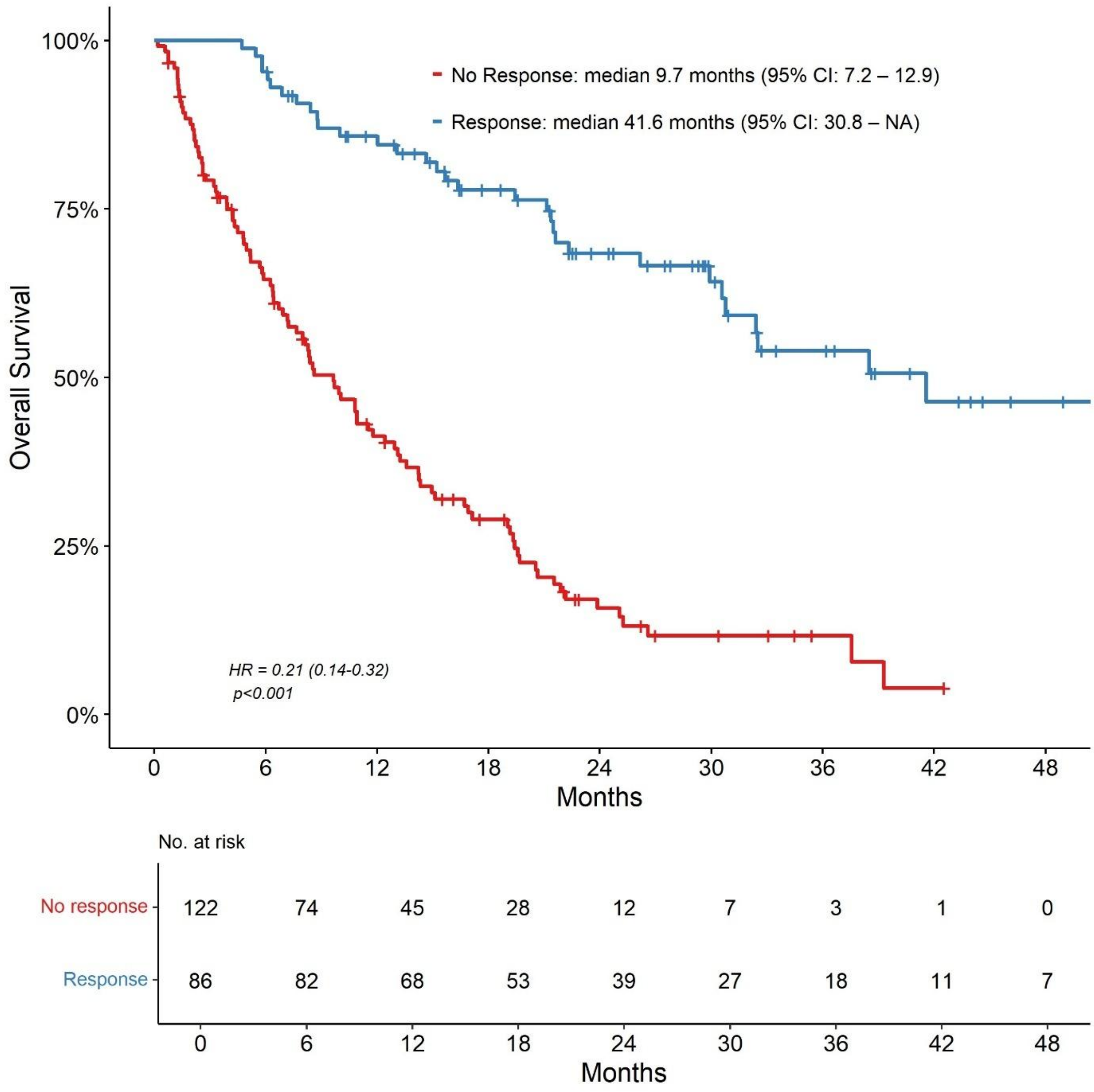

Fig. S4. Survival outcomes in SQ, CC and SH-HCC in the discovery cohort

A, B: Progression-Free Survival and Overall Survival in SQ-HCC and NOS-HCC
C, D: Progression-Free Survival and Overall Survival in CC-HCC and NOS-HCC
E, F: Progression-Free Survival and Overall Survival in SH-HCC and NOS-HCC

HRs from univariate Cox regression are displayed with their 95% CI and p-value (Wald test).

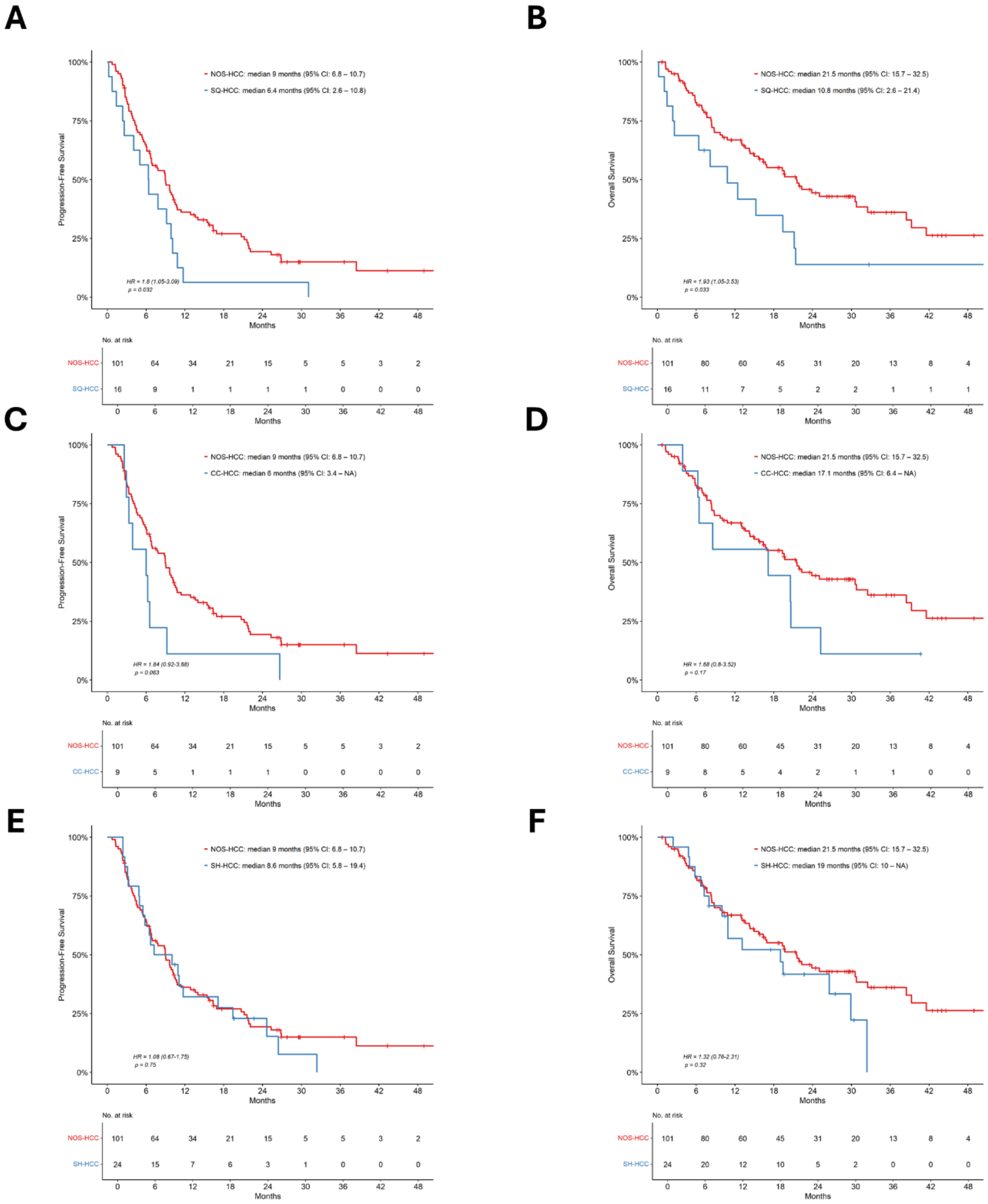

Fig. S5. Representative histological images of the 9 complete VETC-HCC cases diagnosed on HES only from the discovery cohort

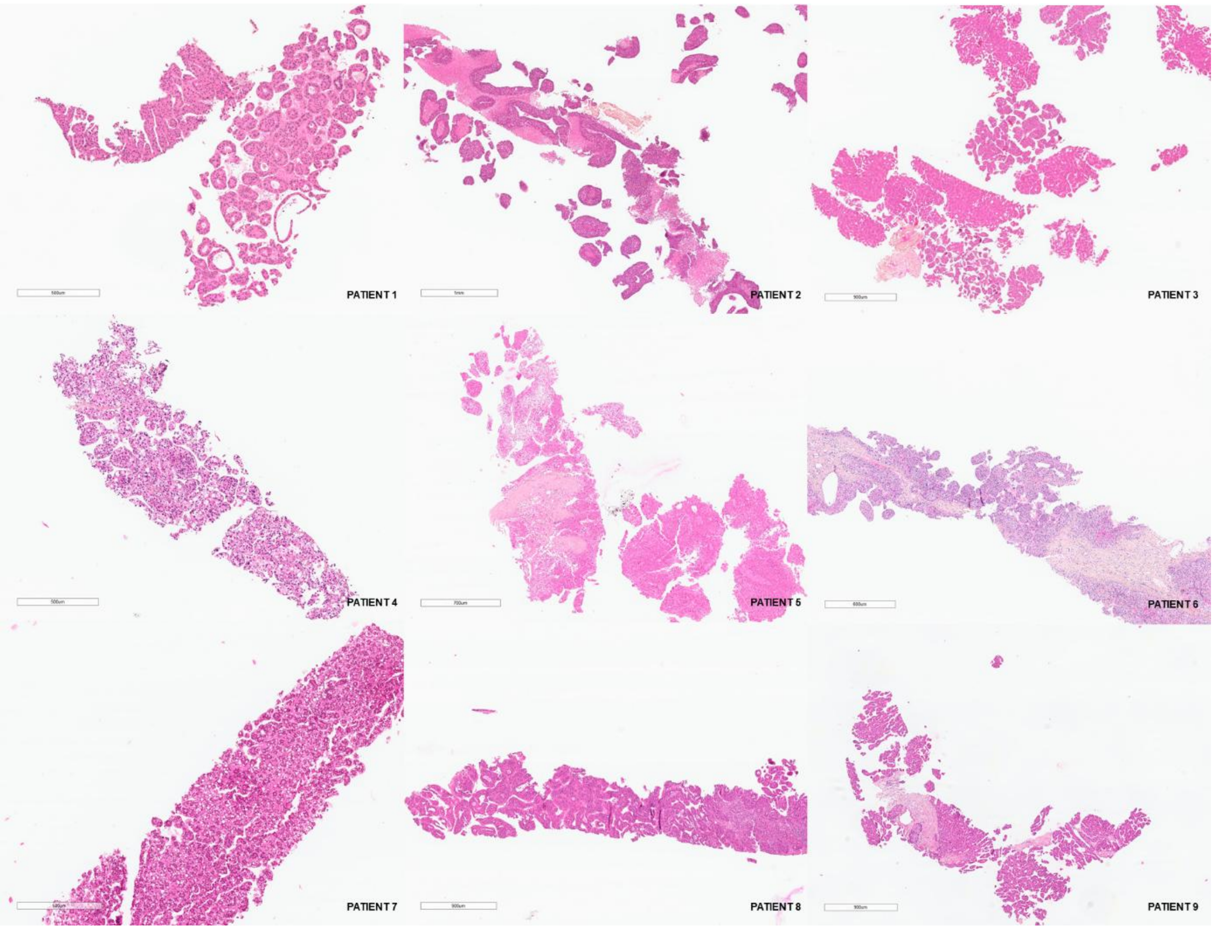

Fig. S6. Survival outcomes in incomplete-VETC-HCC, compared to complete VETC-HCC and non-VETC-HCC in the discovery cohort.

A, B: Progression-Free Survival and Overall Survival in complete VETC-HCC, incomplete-VETC-HCC and non-VETC-HCC.

C, D: Progression-Free Survival and Overall Survival in subgroup of patients who had available CD34 immunostaining, according to VETC phenotype. HRs from univariate Cox regression are displayed with their 95% CI and p-value (Wald test).

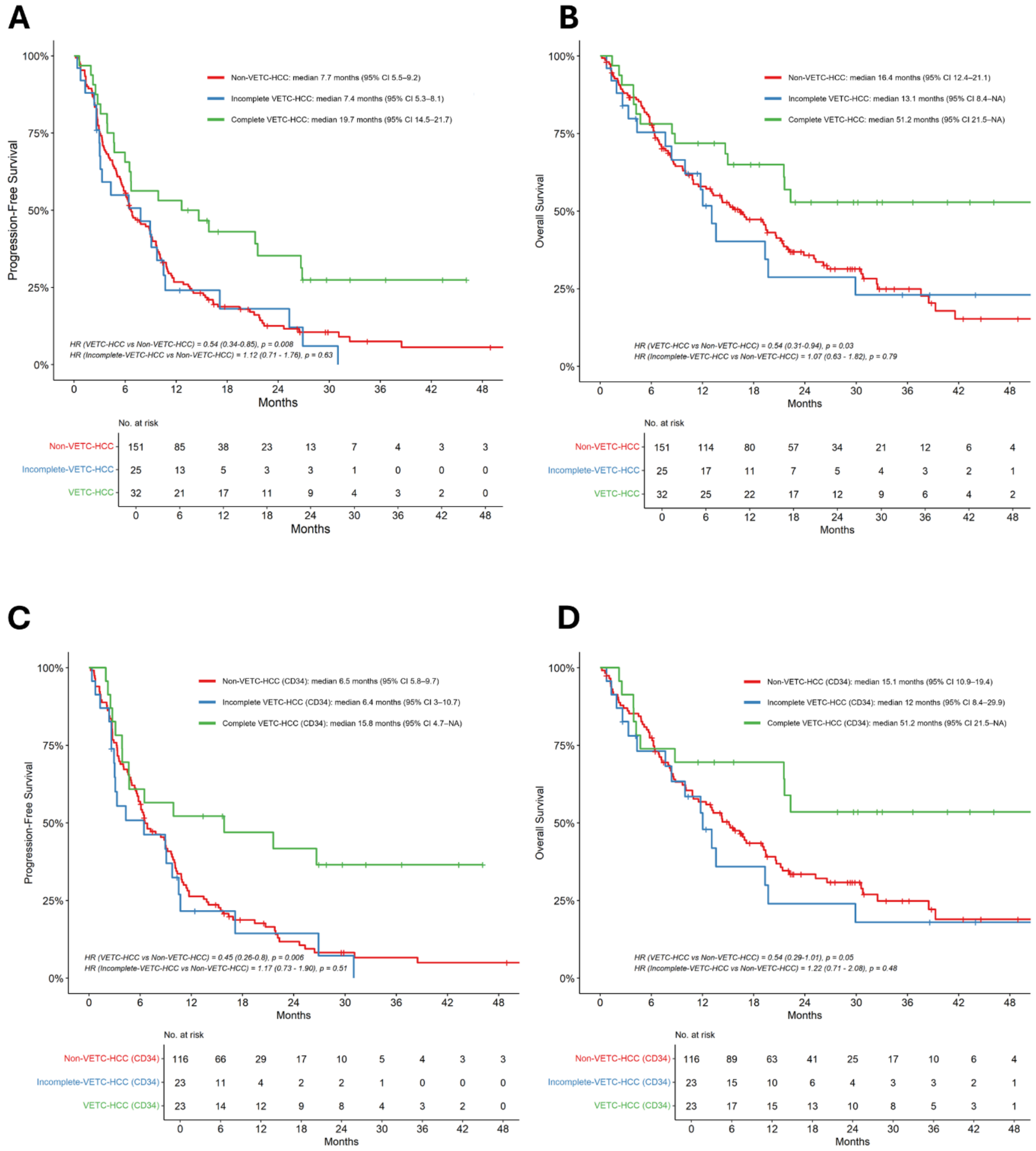

Fig. S7. Effect of VETC threshold on Hazard ratio for Progression-Free survival

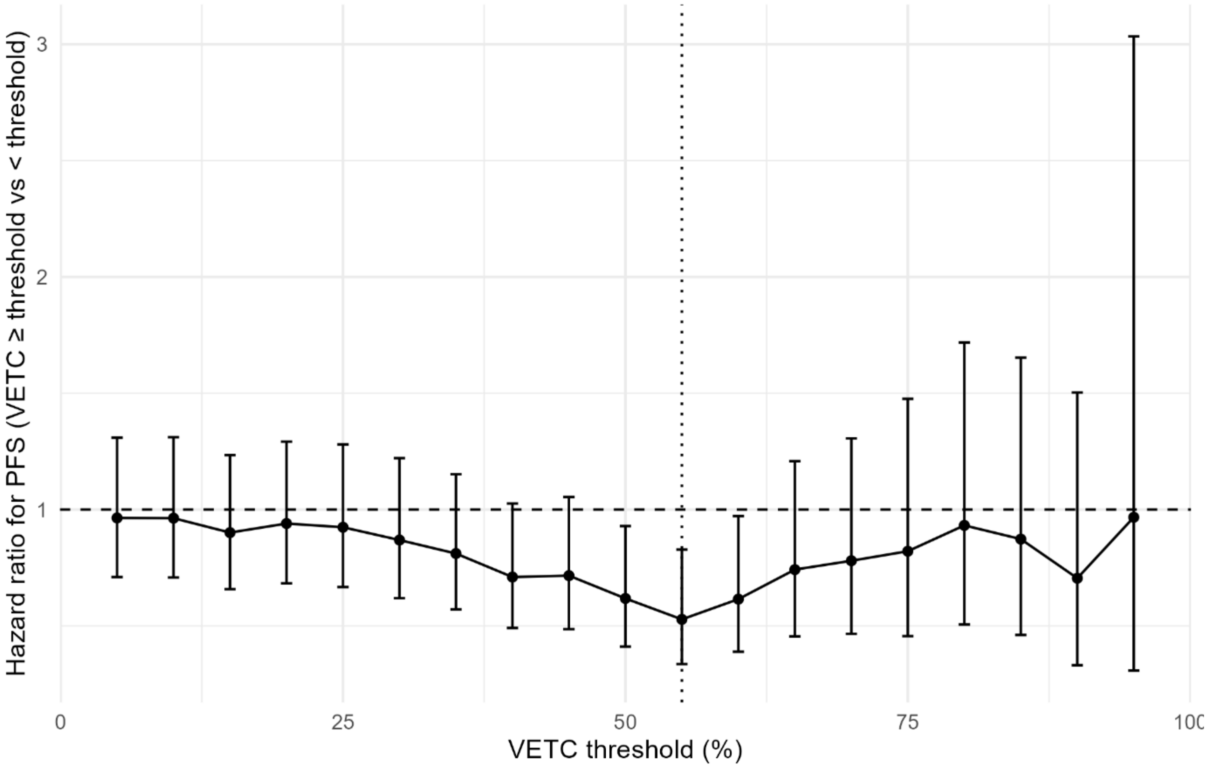

Horizontal dashed line: Hazard Ratio = 1. Vertical dotted line: 55% cut-off for VETC.

Fig. S8. Survival outcomes according to CD8 infiltrate, *TP53* and *CTNNB1* status in patients from Beaujon Hospital in the discovery cohort.

A, B: Progression-Free Survival and Overall Survival in HCC with high or low-CD8 infiltrate

C, D: Progression-Free Survival and Overall Survival according to p53 expression.

E, F: Progression-Free Survival and Overall Survival according to GS/β-catenin expression. HRs from univariate Cox regression are displayed with their 95% CI and p-value (Wald test).

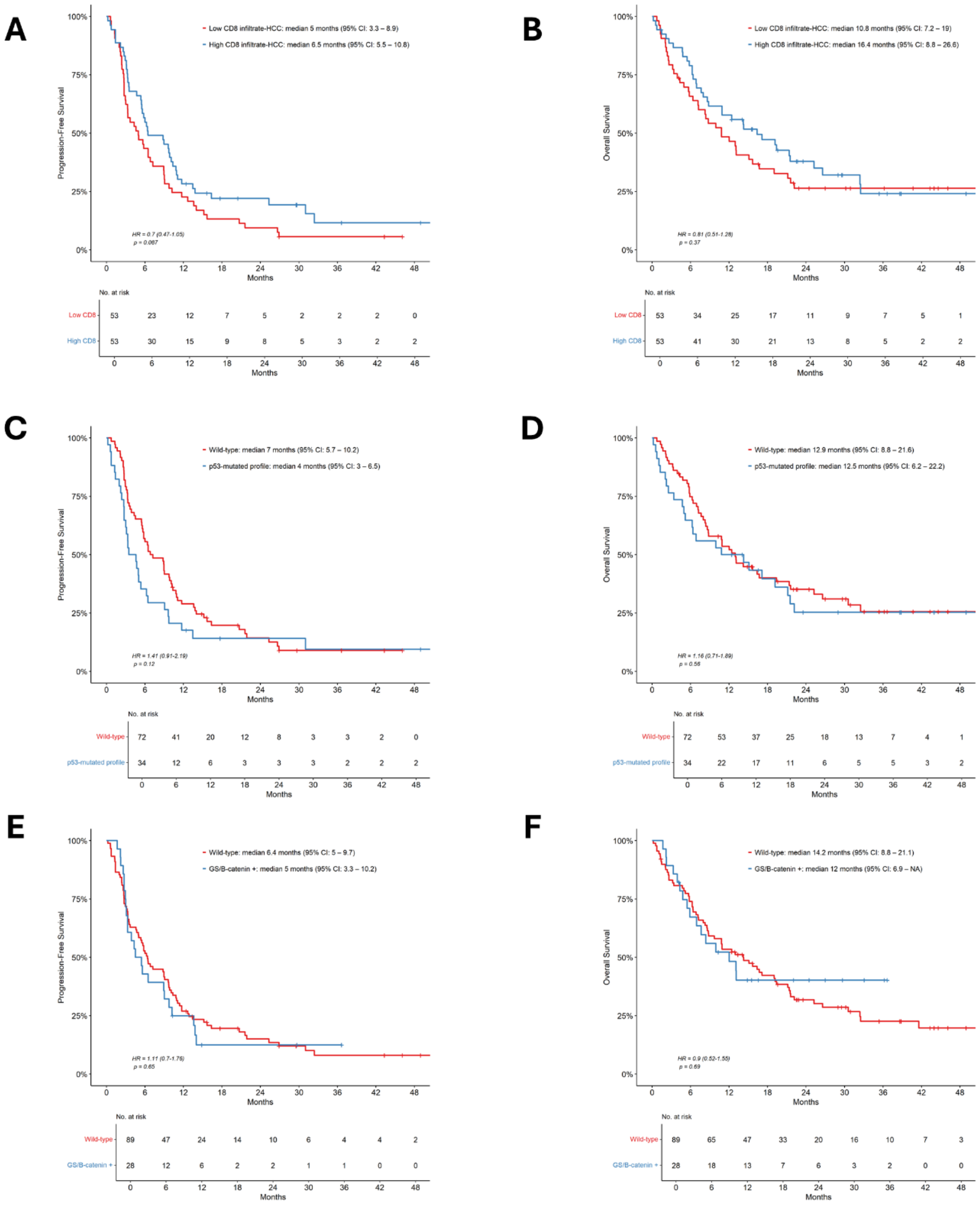

Fig. S9. Predictive impact of atezo+bev on survival of patients with complete VETC HCC (≥55%) in the whole cohort (discovery+validation)

A: Progression-Free Survival according to atezo+bev (red) or durva-treme (blue) treatments in complete VETC-HCC. B: Progression-Free Survival according to atezo+bev (red) or durva-treme (blue) treatments in non or incomplete-VETC-HCC. C: Overall Survival according to atezo+bev (red) or durva-treme (blue) treatments in complete VETC-HCC. D: Overall Survival according to atezo+bev (red) or durva-treme (blue) treatments in non or incomplete-VETC-HCC. HRs from univariate Cox regression are displayed with their 95% CI and p-value (Wald test).

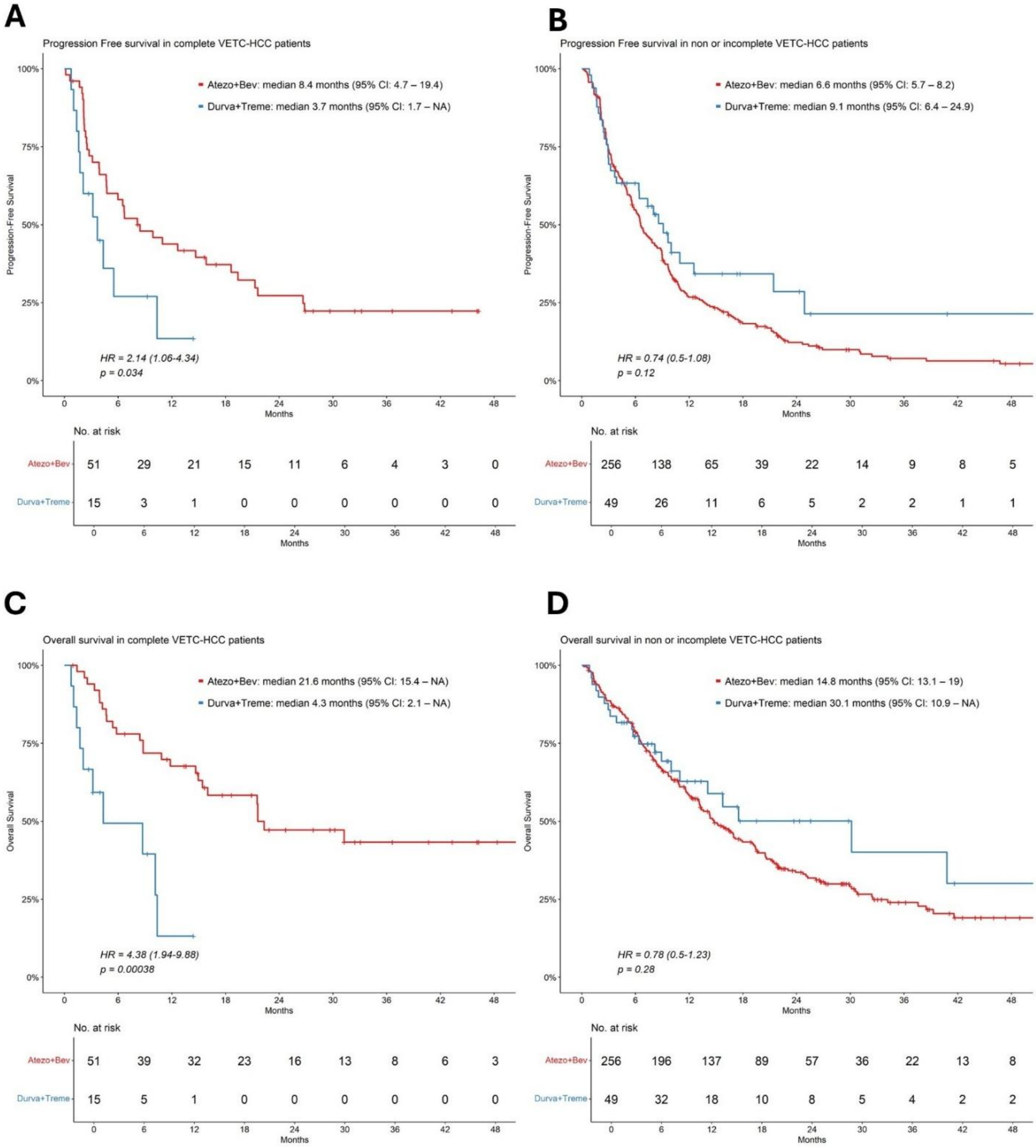

Supplement: Multimedia component 4 [file mmc4.pdf]
